# Heat‐shock‐inducible CRISPR/Cas9 system generates heritable mutations in rice

**DOI:** 10.1002/pld3.145

**Published:** 2019-05-29

**Authors:** Soumen Nandy, Bhuvan Pathak, Shan Zhao, Vibha Srivastava

**Affiliations:** ^1^ Department of Crop, Soil & Environmental Sciences University of Arkansas Fayetteville Arkansas; ^2^ Cell and Molecular Biology Program University of Arkansas Fayetteville Arkansas; ^3^ Department of Horticulture University of Arkansas Fayetteville Arkansas

**Keywords:** CRISPR‐Cas9, genome editing, heat‐inducible expression, heat‐shock promoter, *Oryza sativa*, targeted mutagenesis

## Abstract

Transient expression of CRISPR/Cas9 is an effective approach for limiting its activities and improving its precision in genome editing. Here, we describe the heat‐shock‐inducible CRISPR/Cas9 for controlled genome editing, and demonstrate its efficiency in the model crop, rice. Using the soybean heat‐shock protein gene promoter and the rice *U3* promoter to express Cas9 and sgRNA, respectively, we developed the heat‐shock (HS)‐inducible CRISPR/Cas9 system, and tested its efficacy in targeted mutagenesis. Two loci were targeted in rice, and the presence of targeted mutations was determined before and after the HS treatment. Only a low rate of targeted mutagenesis was detected before HS (~16%), but an increased rate of mutagenesis was observed after the HS treatment among the transgenic lines (50–63%). Analysis of regenerated plants harboring HS‐CRISPR/Cas9 revealed that targeted mutagenesis was suppressed in the plants but induced by HS, which was detectable by Sanger sequencing after a few weeks of HS treatments. Most importantly, the HS‐induced mutations were transmitted to the progeny at a high rate, generating monoallelic and biallelic mutations that independently segregated from the *Cas9* gene. Additionally, off‐target mutations were either undetectable or found at a lower rate in HS‐CRISPR/Cas9 lines as compared to the constitutive‐overexpression CRISPR/Cas9 lines. Taken together, this work shows that HS‐CRISPR/Cas9 is a controlled and reasonably efficient platform for genome editing, and therefore, a promising tool for limiting genome‐wide off‐target effects and improving the precision of genome editing.

**Significance statement**

A method for the temporal control on gene editing based on the use of heat‐shock‐induced expression of CRISPR/Cas9 is described, which was effective in producing heritable mutations in the rice genome. We assume this method will be useful for targeting essential genes and improving the precision of CRISPR/Cas9 platform.

## 1 INTRODUCTION

The CRISPR/Cas9 system is an efficient tool for genome editing that is gaining popularity in both agricultural and medical biotechnology. It consists of two components: the Cas9 nuclease and a single‐guide RNA (sgRNA) that forms a complex (sgRNA:Cas9) and targets sequences complementary to ~20 nt spacer sequence in sgRNA, provided the NGG protospacer adjacent motif (PAM) is located at the *3′* end of the target sequence. Successful targeting by Cas9 results in a blunt double‐stranded break (DSB), 3‐nt upstream of the NGG motif (Cong et al., 2013; Jinek et al., 2012; Mali et al., 2013; Mojica, Díez‐Villaseñor, García‐Martínez, & Almendros, 2009), the repair of which by the cell leads to gene editing effects such as insertion‐deletions (indels) and gene replacement (Jasin & Haber, 2016; Puchta, Dujon, & Hohn, 1996; Rouet, Smih, & Jasin, 1994; Szostak, Orr‐Weaver, Rothstein, & Stahl, 1983; Waterworth, Drury, Bray, & West, 2011). Similarly, CRISPR/Cas12a, an alternative gene editing tool, can be deployed on sequences ending with TTTN motifs (Endo, Masafumi, Kaya, & Toki, 2016; Schindele, Wolter, & Puchta, 2018; Wang, Mao, Lu, Tao, & Zhu, 2017; Zetsche et al., 2015).

To improve the gene editing efficiency, different approaches including sgRNA designs or Cas9 expression systems have been described that mostly include developmental and constitutive gene promoters (Feng et al., 2018; Hu, Meng, Liu, Li, & Wang, 2018; Ma, Zhu, Chen, & Liu, 2016; Miki, Zhang, Zeng, Feng, & Zhu, 2018; Wang et al., 2015). In monocots, rice and maize ubiquitin promoters for Cas9 expression and the *U3* or *U6* promoter for sgRNA expression are quite successful in creating targeted effects in the primary transformed (T0) plants (Lee et al., 2018; Wang et al., 2014; Xie & Yang, 2013). Previous studies have also shown that CRISPR/Cas9 effects could occur at a high rate during tissue culture or regeneration phases, leading to edited T0 lines that efficiently transmit the mutations to the next generation (Mikami, Toki, & Endo, 2015; Srivastava, Underwood, & Zhao, 2017; Zhang, Zhang, Wei, et al., 2014). However, in these approaches, the strong doses of sgRNA:Cas9 could persist far beyond the incidence of targeted gene editing, and provide a wider opportunity to mutagenize the genome‐wide off‐target sites. Accordingly, off‐targeting was found to be higher with the higher doses of sgRNA:Cas9 in human cells, and ~100× higher with constitutive‐Cas9 as compared to the transient‐Cas9 in maize cells, as well as in the rice plants expressing constitutive‐Cas9 (Hsu et al., 2013; Hu et al., 2018; Pattanayak et al., 2013; Svitashev et al., 2015). The dose of the sgRNA:Cas9 complex determines targeting efficiency; however, since mismatches between the sgRNA spacer sequence and the target genomic sites are allowed at the PAM‐distal end (Fu et al., 2013; Jinek et al., 2012; Lin et al., 2014; Liu et al., 2016), each sgRNA could potentially target numerous off‐sites in the genome. Although, off‐sites would generally be targeted at lower rates than the bona fide target site, constitutive or tissue‐specific expression systems would be more permissive to the off‐site mutations by providing strong doses of Cas9 for a longer than necessary period of time.

Off‐target effects of CRISPR/Cas9 are topic of intense investigation as it can induce high‐frequency mutations at unintended off‐target sites. Although, genetic segregation is an option for removing such mutations in many plant species, curbing off‐target effects will be a better approach for developing high‐quality edited lines. Restricted expression of the Cas9 can minimize the off‐target effects while inducing high‐efficiency on‐target mutations. Several approaches for improving the precision of gene editing have been described, for example, high fidelity Cas9, split‐Cas9, and ribonucleoprotein (RNP) Cas9 (Kleinstiver et al., 2016; Liang et al., 2017; Murovec, Guček, Bohanec, Avbelj, & Jerala, 2018; Senturk et al., 2017; Svitashev, Schwartz, Lenderts, Young, & Cigan, 2016; Wright et al., 2015). The use of RNPs has additional benefits in plant biotechnology as this DNA‐free approach generates targeted mutations without incorporating the foreign genes (Wolt, Wang, Sashital, & Lawrence‐Dill, 2016; Wolter & Puchta, 2017). However, RNP approach in plants is faced with the difficulty of delivering the reagent in the cell wall bound compartments, and recovering the edited lines without selection in the tissue culture.

Here, we describe the use of the inducible expression system for controlling CRISPR/Cas9 mutagenesis. Our rationale is to generate short phases of Cas9 expression in the tissue culture or the regenerated plants for allowing targeted genome editing but keeping the Cas9 suppressed at most other times until genetic segregation. In addition to helping reduce off‐target effects, this temporal control on Cas9 could improve gene editing efficiencies by inducing Cas9 in the phases conducive to gene editing, for example, plant regeneration phase in the tissue culture (Srivastava et al., 2017; Zhang, Zhang, Wei, et al., 2014), and enable conditional targeting to avoid lethal effects of mutations.

Using the heat‐shock‐inducible promoter to express Cas9 and the rice *U3* promoter for sgRNAs, we developed transformed lines of rice that essentially contained heat‐shock (HS)‐controlled CRISPR/Cas9 system. By targeting genomic loci with a paired sgRNA, we determined the efficacy and efficiency of HS‐CRISPR/Cas9 system in rice. Our analysis indicates that HS‐CRISPR/Cas9 rarely induced mutations at the ambient room temperatures but efficiently created mutations upon the heat‐shock treatment in the callus and the regenerated plants. Notably, targeted mutations were transmitted to the progeny at a high rate and segregated independently from the *Cas9* gene. In comparison with strong constitutive expression system consisting of the rice Ubiquitin promoter (*RUBI*) to express Cas9 (Xie, Minkenberg, & Yang, 2015), HS‐CRISPR/Cas9 created mutations at ≥50% rate. More importantly, a comparative analysis of the predicated off‐target sites of the designed sgRNAs using the Sanger sequencing showed a higher rate of off‐targeting under constitutive expression system (RUBI), and undetectable and or a lower rate of off‐targeting in the inducible expression system (HS). Overall, this study shows that HS‐CRISPR/Cas9 is a more precise and efficient system for creating targeted mutagenesis, and therefore, a promising platform of improving gene editing that would be less permissive to off‐target effects.

## 2 EXPERIMENTAL PROCEDURES

### 2.1 DNA constructs and plant transformation

**Figure 1:**
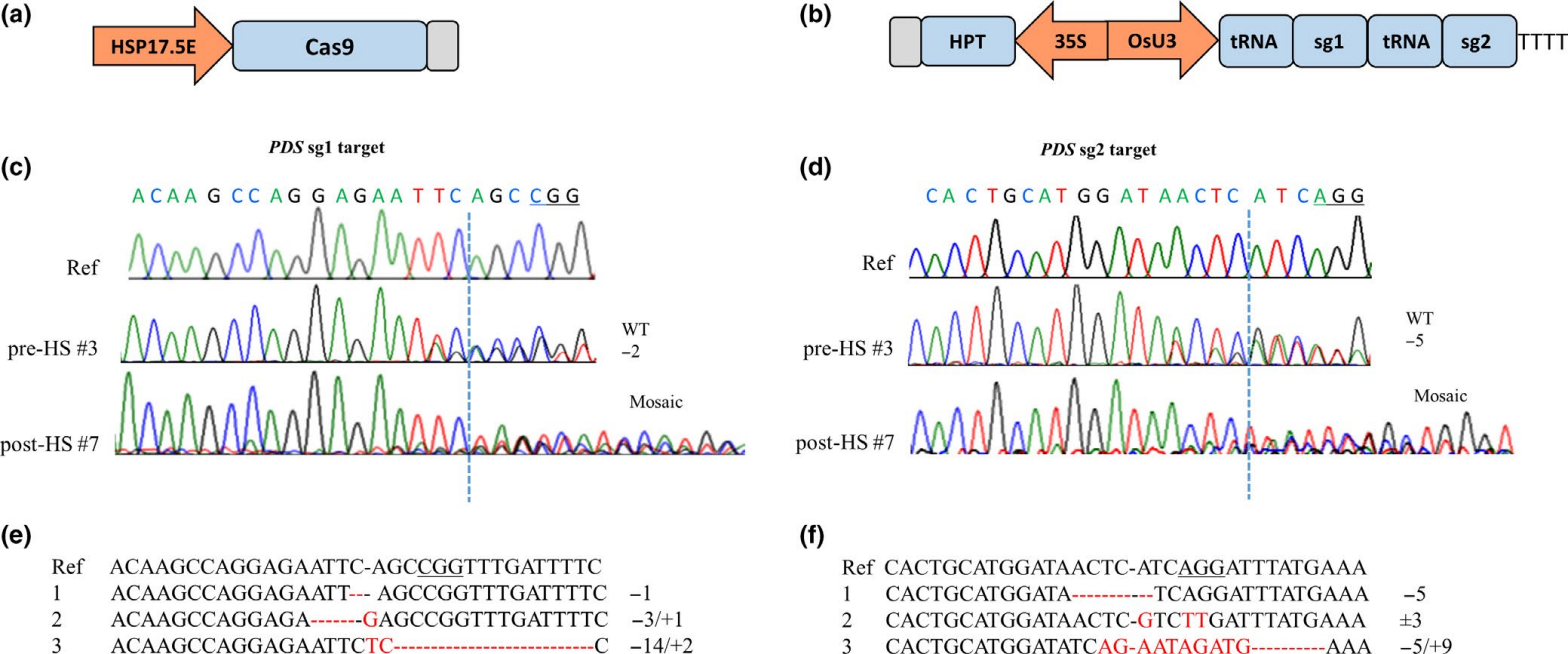
Efficacy of heat‐shock (HS)‐inducible CRISPR/Cas9 on the rice *Phytoene Desaturase* (*PDS*) gene. (a) HS‐Cas9 expression construct consisting of the soybean heat‐shock protein 17.5E (HSP17.5E) gene promoter and the *Streptococcus pyogenes* Cas9 coding sequence; (b) standard sgRNA construct consisting of the rice sno *U3* promoter expressing a pair of sgRNAs via the tRNA processing mechanism. For the plant selection, hygromycin resistance gene consisting of the 35S promoter and the hygromycin phosphotransferase (HPT) gene was included in the construct. Pol III terminator is shown as TTT, and gray bars represent *nos 3′* terminators; (c–d) Sequencing spectra of the *PDS* target sites (PAM underlined) in the wild type reference, and the representative HS‐CRISPR/Cas9‐transformed callus lines, without heat‐shock (pre‐HS) or after a few days of HS (post‐HS). Targeted mutations are indicated by two or multiple overlapping sequence traces (mosaic) near the predicted double‐stranded break (DSB) site (dotted line) in the spectra; (e‐f) Alignments of the reference sequence with the mutant reads as identified by the CRISP‐ID tool or TA cloning. Insertion‐deletions (indels) are indicated by the red fonts and the dashed lines. Number of insertions or deletions is also indicated. PAM site (underlined) and predicted DSB sites (‐) are indicated in the reference sequences

The *Cas9* coding sequence was PCR amplified from pRGE32 (Addgene #63159) using primers (Table S8) laced with specific restriction enzyme sites and cloned between the soybean *HSP17.5E* gene promoter (GenBank accession no. M28070) and the nopaline synthase terminator (*nos 3′*) in the pUC19 vector backbone. The sgRNA vectors were made in pRGE32 backbone using the protocol of Xie et al. (2015) and the sgRNA spacer sequences were selected using the CRISPR RGEN tool (http://www.rgenome.net/cas-designer; Park, Bae, & Kim, 2015). The resulting *GUS* (GenBank accession no. AF485783) and *OsPDS* (Os03g08570) sgRNA constructs were PCR amplified with primers shown in Table S5 and cloned into a vector harboring the 35S promoter driven hygromycin phosphotransferase (*HPT*) gene. All vectors were verified by sequencing. The B1 transgenic line (*cv*. Nipponbare), which has been described by Nandy and Srivastava (2012) or wild type Nipponabare was used for transformation. B1 contains a single‐copy of *GUS* gene controlled by the maize ubiquitin‐1 gene promoter. The GUS activity was verified by staining endosperms using the GUS staining solution described by Jefferson (1987). The embryogenic callus obtained from the mature seeds of the homozygous B1 line was used for all transformations. All transformations were done by the gene gun (PDS1000, Bio‐Rad Inc.)‐based DNA delivery of the Cas9 and the sgRNA vectors (Fig. 1a). The transformed calli were isolated on the hygromycin (50 mg/L)‐containing media. All tissue culture and regeneration in this study were done using the method of Nishimura, Aichi, and Matsuoka (2006).

### 2.2 Heat‐shock treatments

Freshly plated calli, rooted regenerated plants in the glass tubes or ~1‐week‐old seedlings on MS/2 plates were subjected to the heat‐shock (HS) treatment by transferring them to preheated 42°C incubator. The Petri dishes containing the calli or germinating seedlings were laid on their sides between the preheated metal plates, whereas, regenerated plants in the glass tubes were submerged in 42°C water bath. After 3 h, plates or tubes were returned to the tissue culture chamber set at 25°C for further growth. Tissues were harvested after a few days for genotyping by PCR and sequencing.

### 2.3 DNA extraction, PCR, and sequencing

Genomic DNA isolated from callus, regenerated plants or seedlings was used for the polymerase chain reaction (PCR) using primers spanning the target sites (Table S8) or the predicted off‐target sites (Table S9). PCR products were resolved on the agarose gel and extracted using GeneJET Gel Extraction Kit (Thermo Scientific, USA) for sequencing from both ends using the forward and the reverse primers by the Sanger Sequencing method at Eurofins Genomics USA (www.eurofinsgenomics.com). Selected PCR amplicons were cloned into pCR2.1 vector using the TA cloning kit (Thermo‐Fisher Scientific, NY) as per the manufacturer's instructions. Randomly picked 15 to 20 colonies were verified for the insert by PCR using the amplicon‐specific primers and sequenced at Eurofins Genomics USA. The sequence traces (ABI files) were analyzed on the Sequence Scanner 2 software (Applied Biosystems Inc.) and aligned with the reference sequences using the CLUSTAL‐Omega multiple sequence alignment tool. The overlapping sequence traces arising from heterozygous alleles or chimeric samples were separated using the CRISP‐ID tool (Dehairs, Talebi, Cherifi, & Swinnen, 2016).

### 2.4 Gene expression analysis

Young developing leaves were collected from the same tiller and incubated at the room temperature (25°C) or 42°C for 3 h for the control and the heat‐shock treatments, respectively. The total RNA was isolated from 100 mg samples using the QIAGEN RNeasy plant mini kit (Qiagen, Valencia, CA), and treated with RNase‐Free RQ1 DNase (Promega, San Luis Obispo, CA), and quantified using NanoDrop 2000 (Thermo Fisher Scientific, NY). The expression analysis on Cas9 and sgRNAs was performed on 25 ng of RNA using Superscript III Platinum SYBR green one step qRT‐PCR (Life Technologies, Grand Island, NY) in the CFX96 Real‐Time PCR Detection system (Bio‐Rad, Hercules, CA). The values were normalized against the rice ubiquitin gene, and the relative expression to the non‐transgenic control was calculated using the 2^ΔΔCt^ (Livak & Schmittgen, 2001) method. Standard errors of two to six biological replicates were calculated. Each biological replicate was repeated two times for the analysis. Student *t* test (unpaired) was used to determine the *p*‐value. Primers used in qRT‐PCR are given in Table S6.

### 2.5 Off‐target analysis

Potential off‐target sites (OT) for the designed sgRNAs of *GUS* and *PDS* genes were searched using the GGGenome (https://gggenome.dbcls.jp/, Naito, Hino, Bono, & Ui‐Tei, 2015) and the CCTOP (https://crispr.cos.uni-heidelberg.de/; Stemmer, Thumberger, del Sol Keyer, Wittbrodt, & Mateo, 2015) programs with the search queries of 20nt, 12nt seed sequences and ≤4 mismatches. A total of 26 sites for the *GUS* and 30 sites for the *PDS* were shortlisted. The BLAST analysis on all of the 56 sites was performed in the Plant Ensembl and NCBI against *Oryza sativa* Japonica IRGSP 1.0 to verify the sequences and locate their positions (*i.e*. intergenic or genic). Based on (i) the sequence homology across the genome and (ii) the presence/absence of SNPs and/or indels at the off‐target and its surrounding primer designing area; 14 sites for *GUS* and 15 sites for *PDS* sgRNAs were selected for the analysis. The primers flanking the off‐target sites were designed using the Primer Quest tool (https://www.idtdna.com/PrimerQuest/). The primer sequences are shown in Table S9. The PCR was first performed on the negative controls; the WT Nipponbare (*PDS*) and the B1 line (Nipponbare) (*GUS*) and were sequenced by the Sanger method. All the samples were sequenced at Eurofins Genomics USA. The sequence traces were analyzed on Sequence Scanner 2 and aligned with the negative control sequences and the chromosomal reference using the Clustal Omega and t‐coffee multiple sequence alignment tools. The overlapping sequences arising from the heterozygous or chimeric samples were separated using the CRISP‐ID (Dehairs et al., 2016) and Polypeak Parser tools (Hill et al., 2014).

## 3 RESULTS

### 3.1 Heat‐shock‐induced CRISPR/Cas9 mutagenesis in the rice *in vitro* tissue

**Figure 2:**
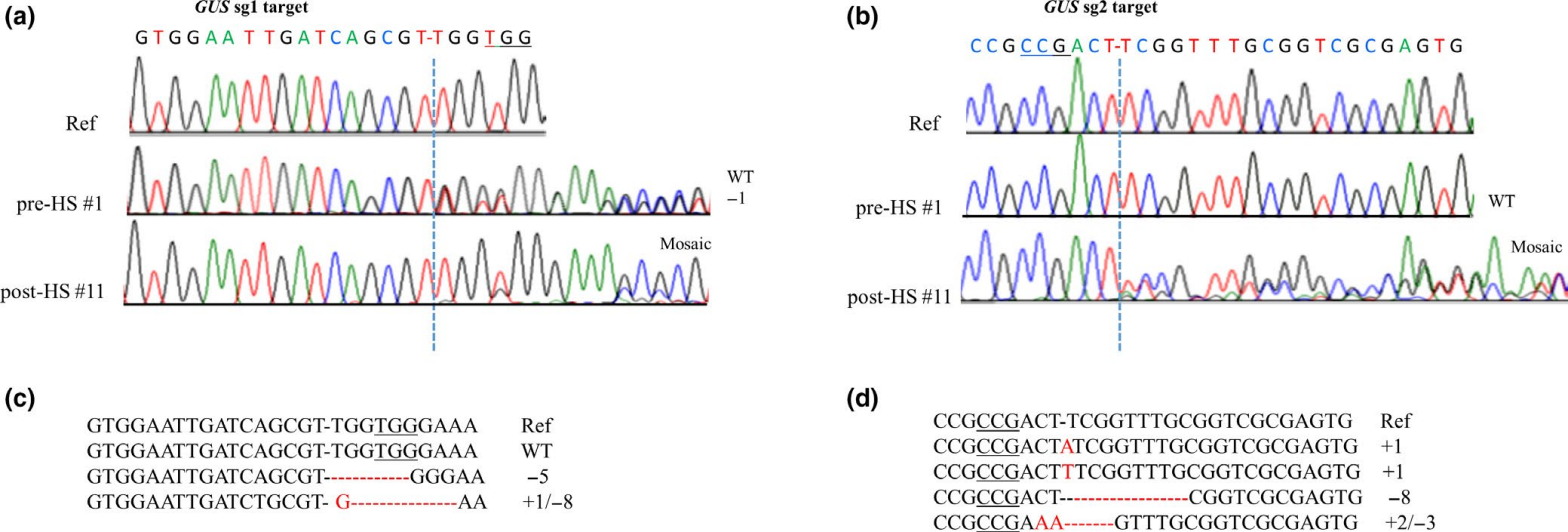
Efficacy of HS‐CRISPR/Cas9 on the *GUS* transgene located in the rice genome. (a, b) Sequencing spectra of the *GUS* target sequences from the parental B1 line (ref., PAM underlined), and the targeted callus lines, without heat‐shock (pre‐HS) or with HS treatment (post‐HS). Dotted vertical lines represent the predicted DSB sites. Overlapping sequence traces in the spectra indicate the mosaic mutation pattern; (c, d) Mutations in the spectra as identified by the CRISP‐ID tool (c) or TA cloning (d). Dashes indicate deletions, and the red letters indicate insertions. Number of insertions‐deletions in each sequence is indicated. PAM site (underlined) and the predicted DSB sites (‐) are also indicated

We used the soybean heat‐shock protein 17.5E (*HSP17.5E*) gene promoter to express the humanized *Streptococcus pyogenes* Cas9 (SpCas9), and the tRNA‐processing system to express two sgRNAs by the rice snoRNA *U3* promoter (Czarnecka, Ingersoll, & Gurley, 1992; Xie et al., 2015; Fig. 1a,b). The motivation to use *HSP17.5E* promoter was based on its observed efficacy in controlling the Cre‐*lox* recombination in the tissue culture‐derived rice plants and seedlings. Earlier, we showed that a simple heat treatment of 42°C for 3 h led to efficient Cre‐*lox*‐mediated excision of the marker gene in rice seedlings and inheritance of the marker‐free locus by their progeny (Nandy & Srivastava, 2012). We chose previously tested target loci and sgRNAs for this study that include rice *Phytoene Desaturase* gene (*OsPDS*) and the β‐*Glucuronidase* transgene inserted in the rice genome (Srivastava et al., 2017). For *GUS* targeting, a well‐characterized transgenic line, B1 (cv. Nipponbare), that harbors a single‐copy of the *GUS* gene driven by the maize ubiquitin promoter (*Ubi*), and for *PDS* targeting, non‐transgenic Nipponbare was transformed. The resulting hygromycin‐resistant calli were maintained and regenerated at the ambient room temperature. For testing HS‐CRISPR/Cas9 activity, randomly sampled calli were either kept at the room temperature (pre‐HS) or transferred to the fresh media plate for heat‐shock treatment, and analyzed 5–7 days later (post‐HS). A total of 23 PDS and 12 GUS calli were screened for mutations at the two sgRNA sites (Table 1). Two out of the 12 pre‐HS PDS calli were found to contain the targeted mutations, one of which contained monoallelic mutation at both sg sites, while the other showed biallelic heterozygous mutation at the sg2 site (Table S1). Similarly, one of the 6 pre‐HS GUS samples showed mutations (monoallelic) at the sg1 target (Table 1; Table S2). The pre‐HS mutations could be derived from the leaky HS‐Cas9 activity and established early in the selection of the transformed clones. Accordingly, characteristic overlapping dual traces were observed in the pre‐HS samples, representing heterozygous or chimeric clones (Figs 1c,d, 2a,b). Next, the calli were subjected to heat‐shock (HS) treatment for 3 h and returned to ambient room temperature for further growth. After 5–7 days (post‐HS), freshly grown tissue from each callus culture was analyzed. Since calli could contain multiple independent mutations, HS‐induced targeting could contain multiple overlapping traces in the Sanger sequencing spectra downstream of the predicted DSB sites (Fig. 1c,d). Further, if induced mutations are rare in the post‐HS samples, they would appear as the minor trace in the sequencing spectra (Fig. 2a,b). Accordingly, overlapping and/or minor traces in the sequencing spectra were found in 7 *PDS* and 3 GUS calli, indicating mosaic pattern of mutations due to HS‐CRISPR/Cas9 activity (Table 1; Tables S1–S2). Mosaic pattern was observed at PDS sg1 site in 3 samples and at PDS sg2 site in 7 samples (Table S1). Similarly, mosaic pattern in GUS samples occurred once in the sg1 site and three times in the GUS sg2 site (Table S2). In summary, HS‐CRISPR/Cas9 was effective in creating targeted mutations with a higher rate of targeting in post‐HS calli (50–63%) as compared to the pre‐HS calli (16%) of rice (Table 1). To verify these mutations, traces were separated using the CRISP‐ID tool or subjected to TA cloning and colony sequencing. These analyses revealed indels at the predicted DSB sites, indicating CRISPR/Cas9 mediated mutagenesis (Fig. 1e–f, 2c,d). In conclusion, *HSP17.5E‐Cas9* is effective in creating induced targeted mutations in the rice calli. With the paired sgRNAs, HS‐CRISPR/Cas9 generated HS‐induced mutations in ≥50% of the transformants (Table 1). All callus cultures were subjected to plant regeneration; however, PDS cultures mostly appeared non‐embryogenic, while GUS cultures regenerated plants. Therefore, all subsequent work was done with HS‐CRISPR/Cas9 targeting the *GUS* transgene.

**Table 1:** HS‐CRISPR/Cas9 activity in rice callus

Exp.	Target	Total no. of calli	Pre‐HS calli^a^			Post‐HS calli^a^		
			Total no.	Targeted^b^	Eff.^c^	Total no.	Targeted^b^	Eff.^c^
1	*PDS*	23	12	2	16	11	7	63.6
2	*GUS*	12	6	1	16	6	3	50.0

a Number of room temperature (pre‐HS) or heat‐shocked (post‐HS) calli showing mutations at the two (sg1, sg2) target sites.

b Indels at DSB sites of sg1 or sg2 targets.

c Percent calli showing targeted mutations at one or both targets. See Tables S1 and S2 for description of each line analyzed.

### 3.2 Heat‐shock‐induced targeting in T0 plants

**Figure 3:**
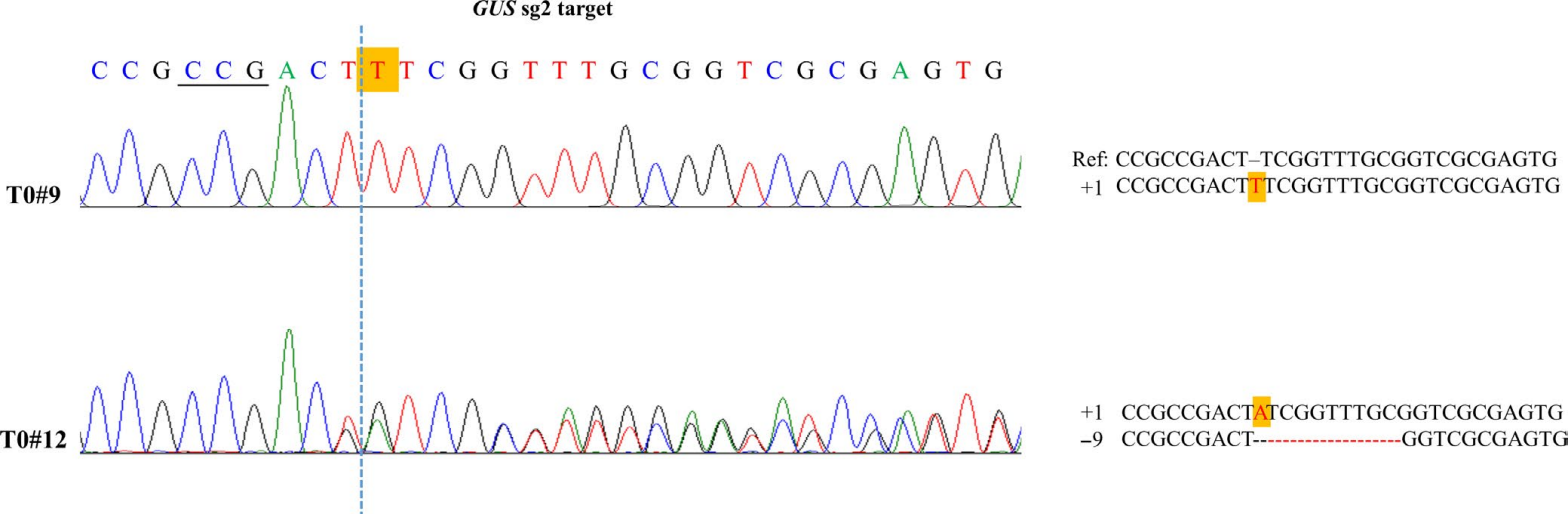
Sequencing of the *GUS* sg2 target site in T0 plants #9 and #12 harboring HS‐CRISPR/Cas9 constructs. Mutation types are shown adjacent to each spectrum along with the reference sequence. Dashed vertical line indicates the predicted DSB site. PAM site is underlined. Shaded red letter indicates insertions, and dashes indicate deletions. The two sequences in T0#12 were separated using the CRISP ID tool

**Figure 4:**
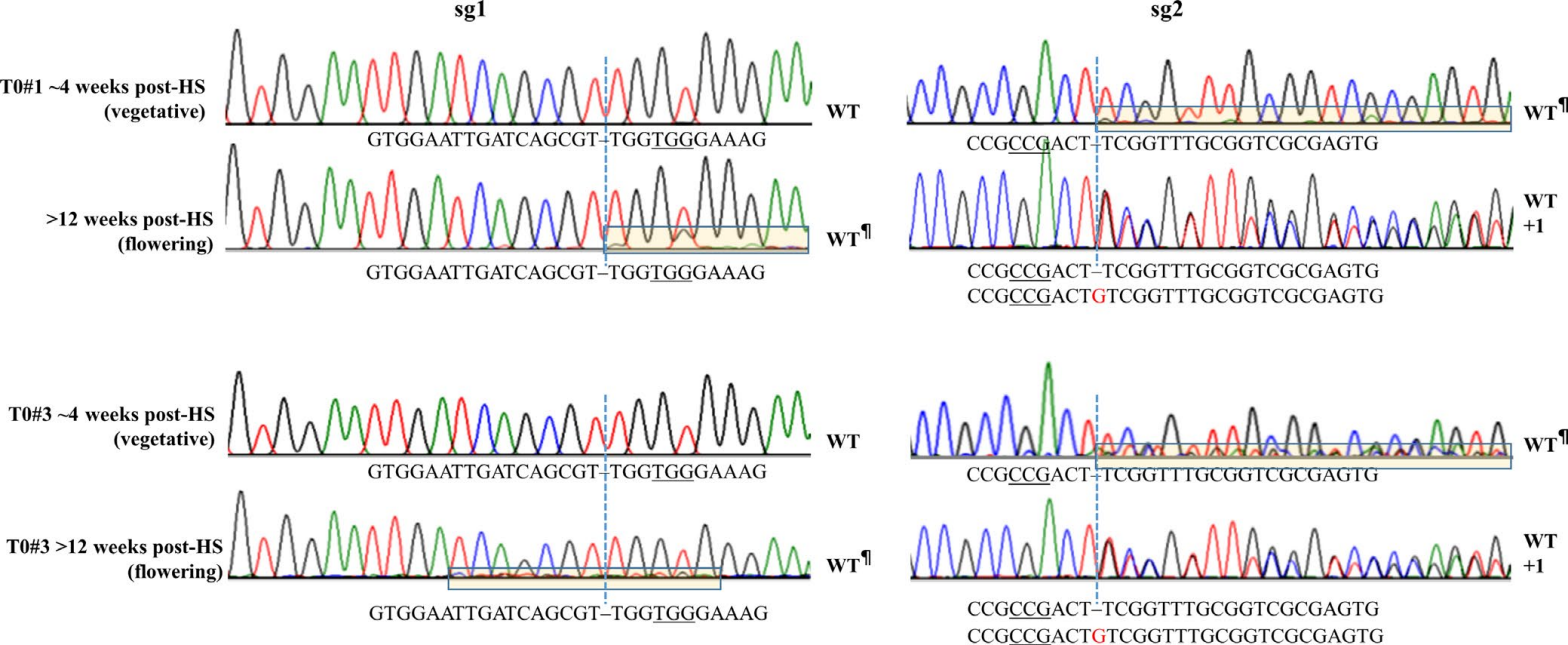
Genotyping of T0 plants #1 (a) and #3 (b) at *GUS* sg1 and sg2 sites by PCR‐sequencing at two growth stages, ~4 weeks after heat‐shock (HS) or the vegetative stage and ~12 weeks after HS or the flowering stage. Mutation types are shown below each sequencing spectra with the PAM sequence underlined. The predicted DSB sites are indicated by the vertical lines. The baseline secondary sequence traces in the spectra are boxed, indicating a low rate of mutations in largely wild type samples (WT^¶^; see Table 2). The spectra containing two overlapping sequences were analyzed by the CRISP‐ID tool to identify monoallelic +1 mutations in the two plants. Major sequences in the remaining are shown below each spectrum

**Figure 5:**
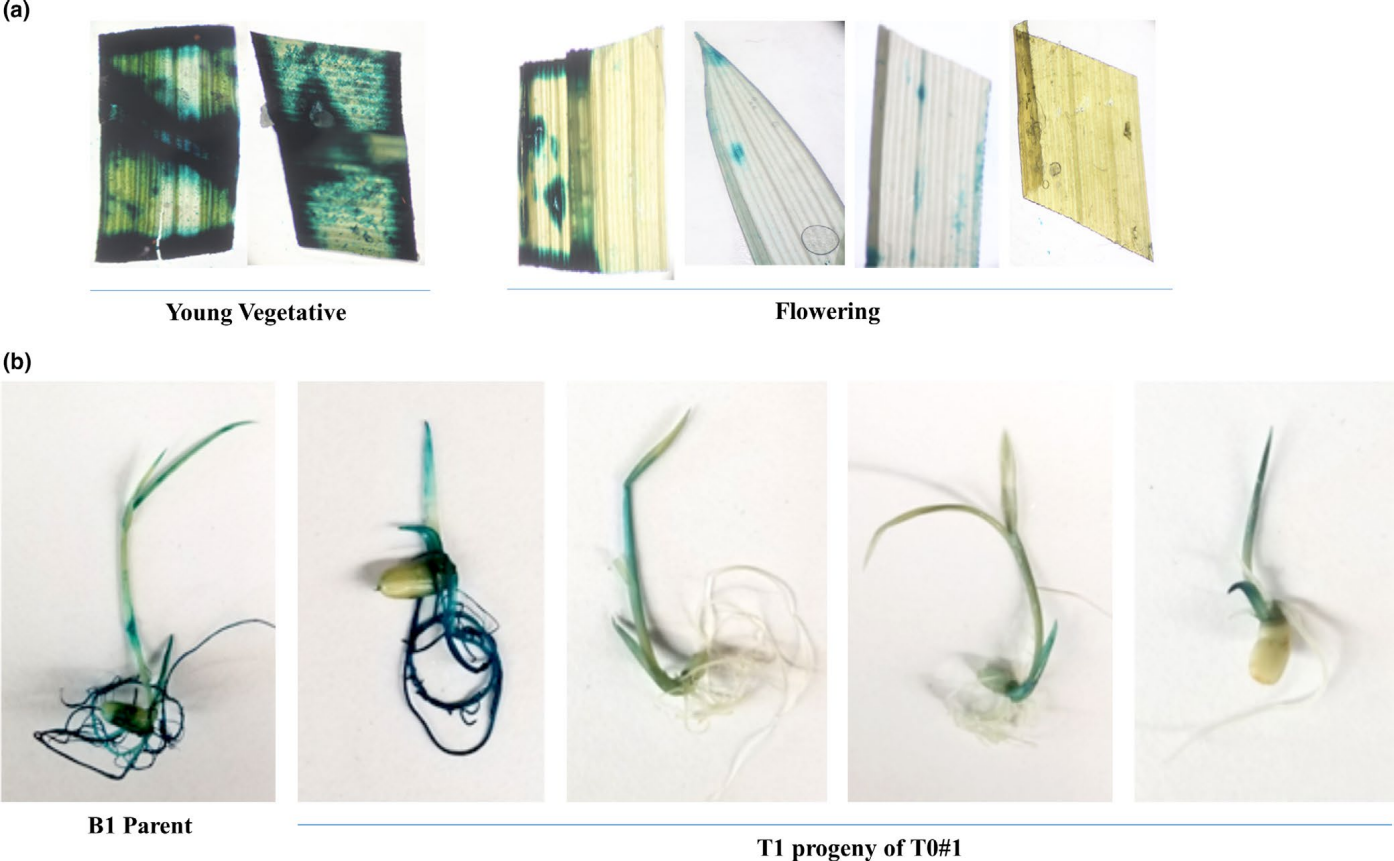
Histochemical GUS staining in the HS‐CRISPR/Cas9 line. (a) Leaf cuttings from the post‐HS T0#1 plant at the young vegetative stage and from the flowering plant. Note the staining in the cut end and poked points, and diminished staining in the leaves of flowering plant; (b) Seedlings of the control B1 line harboring the GUS gene and the progeny of the HS‐CRISPR/Cas9 line #1

Twenty regenerated plants (T0) expressing HS‐CRISPR/Cas9 against the *GUS* gene were obtained from two experiments. At the rooting stage, 1–3 leaf samples from each were subjected to PCR and Sanger sequencing at the targeted sites. Two of the T0 plants (#9 and #12) were found to harbor homozygous or heterozygous mutations at the sg2 target, indicating leaky pre‐HS Cas9 expression in these plants (Fig. 3). The rest did not show mutations at either site (Table 2). Next, T0 plants were given two rounds of HS treatment by transferring them to 42°C incubator for 3 h and repeating the treatment after ~20 h of rest at the room temperature. The HS plants were subsequently transplanted in the soil and grown in the greenhouse. After ~4 weeks of HS treatment, at the young vegetative stage, target site analysis by PCR and sequencing was conducted in 2–3 leaf samples. No detectable targeting was found in any of the samples except those derived from T0#9 and #12; although, a baseline secondary sequence was detected in the sequencing spectra of a few lines, indicating a low rate of HS‐induced mutations (Table 2). T0#1 and #3 showed a clear WT sg1 target in the young plants but minor targeting, indicated by the secondary baseline sequence trace, in the flowering plant. At the sg2 target, on the other hand, these plants showed minor targeting in the young plants, but monoallelic targeting in the flowering plants (Fig. 4a,b). Similar mixed traces were observed in the other post‐HS samples of different T0 plants (Fig. S1). These observations corroborated with histochemical GUS staining as these plants progressively lost GUS activity. For example, T0#1 showed strong GUS staining in the leaf cuttings taken from the young vegetative plant but diminished staining in the leaves collected from the flowering plant (Fig. 5a; Table 2). Similarly, T0#3 progressively lost GUS activity, while T0#2 that lacked detectable mutations continued to show strong GUS staining, and T0#9 and #12 that harbored biallelic mutations also did not display GUS staining in the leaves derived from the vegetative or flowering stages of the plant (Table 2; Fig. S2). These observations are analogous to our work with HS Cre‐*lox* system, in which, rice seedlings harboring *HS Cre* showed progressive recombination in the heat‐shocked plants, and transmitted the recombined locus to the next generation (Nandy & Srivastava, 2012). Taken together, HS‐induced gene editing effects likely occurred in the early cell lineages and established in the plant through cell division.

**Table 2:** Characterization of T0 Plants transformed with HS‐CRISPR/Cas9 targeting GUS gene

Line	GUS staining^a^		Cas9 expression		Sg1	Sg2	Off‐target studied
	Y	O	Fold‐induction by HS	% RUBI‐Cas9^b^			
1	+	−	7.0	0.03	WT^e^	WT^e^	Yes
2	+	+	0.35^c^	0.07	WT	WT	Yes
3	+	−	2.5	0.13	WT^e^	WT^e^	Yes
4	+	+	10	0.02	WT	WT	–
5	+	+	84	0.03	WT	WT	Yes
6	+	+	–	–	WT	WT	–
7	+	−	–	–	WT^e^	WT^e^	–
8	+	+	–	–	WT	WT	–
9	−	−	–	–	WT	Biallelic	–
10	+	+	0.45^c^	0.2	WT	WT	Yes
11	+	+	–	–	WT	WT	–
12	−	−	63	5.96	WT	Biallelic	Yes
13	+	−	–		WT	WT^e^	–
14	+	+	1^d^	16.96	WT	WT	–
15	+	+	2.2	–	WT	WT	–
16	+	+	–	–	WT	WT	–
17	+	+	–	–	WT	WT	–
18	+	−	6.9	0.09	WT	WT	Yes
19	+	−	9.2	0.02	WT^e^	WT	Yes
20	+	+	3.1	0.03	WT	WT	Yes
RUBI‐1	−	−	–	100	Biallelic	Biallelic	Yes
RUBI‐2	+	+	–	100	–	–	–
RUBI‐3	+	+	–	50	–	–	–

a Histochemical staining of leaf cuttings from young vegetative (Y) or older flowering (O) plants.

b Non‐induced (room temp) expression value in HS‐Cas9 compared to RUBI‐Cas9 expression values.

c Silenced Cas9 lines.

d Overexpression Cas9 lines.

e Baseline secondary sequence trace in the sequencing spectra (see Fig. S1).

T0 plants #1, #2, #3 flowered and set seeds. These plants were analyzed at the flowering stage (>12 weeks post‐HS) for the presence of mutations at the target sites. As shown in Fig. 4a,b, T0#1 and #3 showed rare targeting at the sg1 site but a clear monoallelic targeting at the sg2 site. Since, a low rate of mutagenesis at sg2 was detected in these plants at the young vegetative stage (baseline minor trace in the spectra) (Fig. 4a,b), these monoallelic mutations were likely induced early in the plant. Both plants contained a characteristic + 1 mutation at the predicted DSB site. T0#2, however, did not show mutations in any of analyzed tissue, and later was found to contain a silenced Cas9 gene (described below).

**Figure 6:**
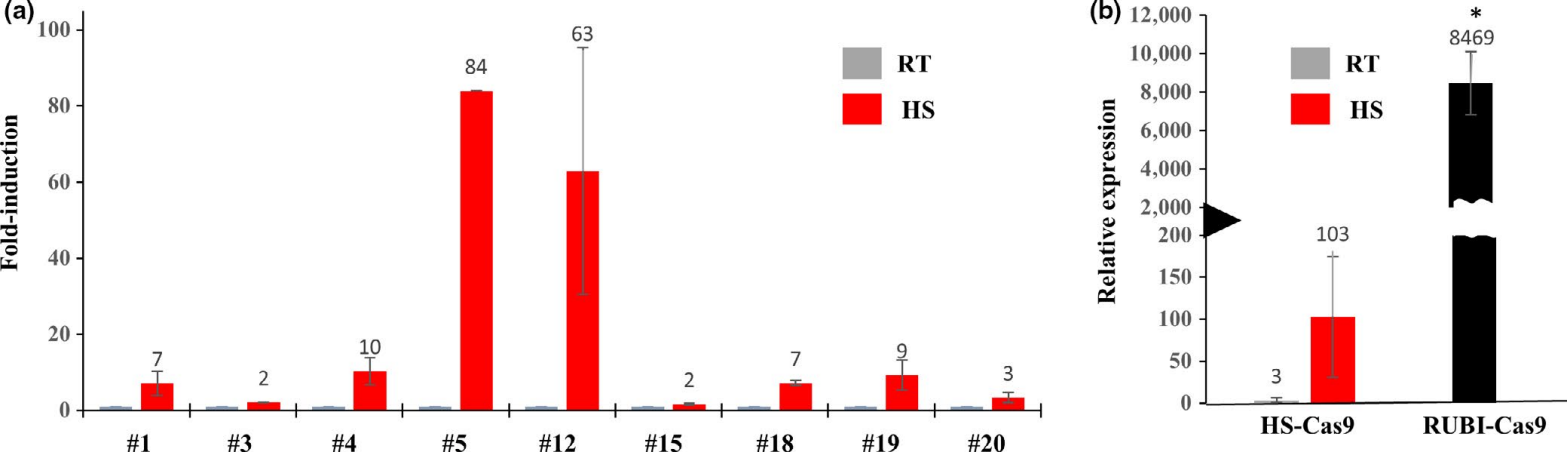
Cas9 expression analysis. (a) Fold‐induction of Cas9 in T0 plants by the heat‐shock (HS) treatment (3 h exposure to 42°C) as compared to the background room‐temperature (RT) values; (b) Relative expression of Cas9 in HS‐Cas9 lines with respect to the constitutive RUBI‐Cas9 lines. The expression in HS‐Cas9 lines was calculated at RT and upon HS. The average of 8 HS‐Cas9 lines and 3 RUBI‐Cas9 lines is shown with standard errors (*p‐value < 0.001)

The Cas9 expression was analyzed in a subset of T0 plants and compared with non‐transgenic wild‐type and the constitutive Cas9 lines using the real‐time quantitative PCR. Of 12 plants, nine showed an increase in the Cas9 expression (2–84×) upon HS over their respective room‐temperature (RT) values (Fig. 6a; Table 2). Two T0 plants (#2, #10) appeared to be silenced as the relative Cas9 expression did not increase by the HS treatment in these plants, whereas #14 showed equally high expression at RT and HS (Table 2). Three constitutive‐Cas9 lines expressing RUBI‐CRISPR/Cas9 (RUBI‐1, 2, 3) were included in the analysis, each of which showed strong relative expression, and one of them (RUBI‐1) harbored targeted mutations in the *GUS* gene (Table 2). In comparison to these RUBI‐Cas9 lines, the Cas9 expression was three orders of magnitude lower in HS‐Cas9 lines, which could be induced ~34‐fold by HS (Fig. 6b; Table 2).

### 3.3 Inheritance of targeted mutations by the progeny

T0#1 and #3 were selected for the progeny analysis. These plants, at the young vegetative stages, showed strong GUS activity but diminished activity in the flowering stages, presumably due to multiplication of cells harboring mutations in the *GUS* gene (Fig. 5a; S2; Table 2). Sequencing of the sg1 and sg2 sites in these plants at the flowering stage detected a rare targeted mutagenesis in the sg1 site and a monoallelic mutation at the sg2 site (Fig. 4a,b).

**Figure 7:**
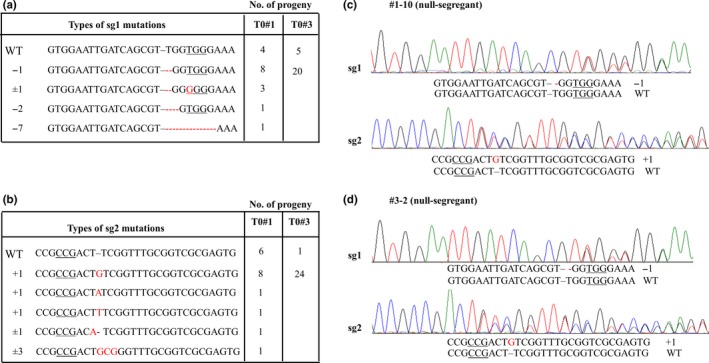
Inheritance of HS‐CRISPR/Cas9‐induced mutations by the progeny of T0#1 and #3. (a, b) Number of T1 plants harboring monoallelic or biallelic indels at the *GUS* sg1 and sg2 target sites. Indels are shown as dashes and the red letters; (c, d) Inheritance of mutations in the two Cas9 null‐segregants harboring monoallelic mutations at the sg1 and sg2 sites. The sequence reads as identified by separating overlapping reads by the CRISP‐ID tool and their alignments are shown below each spectrum. Insertion and deletion are shown by red letter or dashes. PAM is underlined

Twenty‐four seeds derived from T0#1 parent and 30 seeds from T0#3 parent were germinated for the progeny analysis. When their coleoptiles were fully emerged, seedlings were subjected to 2–3 rounds of HS treatment. Therefore, *de novo* targeting could occur in the Cas9+ lines. Histochemical GUS staining of these seedlings (~2 weeks after germination) showed strong (+) or diminished (−) GUS staining (Fig. 5b; Tables S3, S4). As expected, *Cas9* independently segregated in the population, and a few null‐segregants were identified (Table 3). A subset of 16 T1 plants derived from T0#1 was subjected to PCR/sequencing at sg1 and/or sg2 sites. At the sg1 site, 11 contained monoallelic (68.7%) and one biallelic mutations (6.2%), while at sg2 site, nine contained monoallelic (56.2%) and one biallelic (6.2%) mutations (Table 3). Analysis of 25 T0#3 progeny, on the other hand, revealed monoallelic and biallelic mutations at the sg1 site in 18 (72%) and two (8%), respectively, while at sg2 only monoallelic mutations (96%) were found (Table 3). The remaining inherited the WT allele. The analysis of mutant reads revealed 4–5 types of mutations among T0#1 progeny but only one type at each site among T0#3 progeny (Fig. 7a‐b). The abundance of one type of mutation in each population indicates a high rate of inheritance, which was confirmed by three Cas9 null‐segregants in each population that harbored mutations at the sg1 and/or sg2 sites (Fig. 7c,d). The detection of only one type of mutation among T0#3 progeny raises the question whether this line is derived from *HS‐Cas9* activity induced by the tissue culture. However, since the analysis of three different leaf samples of T0#3 plant detected only the WT sg1 site (Fig. 4b), the observed mutations are likely established in the germline at a later stage, possibly after the HS treatment of this plant.

**Table 3:** Inheritance of HS‐CRISPR/Cas9‐induced mutations by the progeny

Parent	No. of T1 plants analyzed	Cas9 (+)	Cas9 (‐)	GUS staining^a^		% Mutants at Sg1		% Mutants at Sg2	
				+	−	Monoallelic	Biallelic	Monoallelic	Biallelic
T0#1	24	18	6	4	20	68.7	6.2	56.2	6.2
T0#3	30	25	5	–	30	72	8	96	–

a Histochemical staining of leaf cuttings showing strong (+) or weak/no (−) staining.

### 3.4 Reduced rate of off‐targeting in HS‐CRISPR/Cas9 lines

A total of 29 off‐target (OT) sites with significant matches to the four designed sgRNAs against *GUS* or *PDS* genes were selected for PCR‐sequencing analysis (Table S5, S6). However, six GUS‐OTs could not be validated by sequencing in the parental controls, and therefore, removed from the analysis. The remaining 23 OTs, representing eight GUS‐OTs and 15 PDS‐OTs, were analyzed in their respective transgenic lines. In order to compare the rates of off‐targeting between the inducible (HS‐Cas9) and the constitutive (RUBI‐Cas9) expression systems, RUBI‐CRISPR/Cas9 lines targeting *PDS* and *GUS* were included in this analysis (Table S7). The only difference between the RUBI‐ and HS‐CRISPR/Cas9 lines used in this study is the promoter of Cas9, while both expressed the same sgRNAs by the rice *U3* promoter.

Four of the 23 OTs, representing the intergenic or intronic regions, were found to be targeted in one or more lines, whereas, targeting in the remaining 19 OTs was undetectable in both RUBI‐ or HS‐Cas9 lines analyzed in this study (Tables S5, S6). Off‐targeting by Cas9 was defined as insertion‐deletions (indels) at the predicted DSB site; although, other effects such as base substitution, and the occasional single base insertion in the seed sequences were also observed (Fig. S3). Only one line showed 3‐nt insertion near PAM but away from DSB of GUS OT‐11. This variation was called as “other effects” since it did not occur at the predicted DSB site. Tissue culture is widely known to induce somaclonal variations, including transitions and transversions in the intergenic and intronic regions at high rates (Tang et al., 2018; Zhang, Wang, et al., 2014). Therefore, the observed single‐nucleotide variations in the seed sequences or PAM that did not fall in the DSB site were called as non‐Cas9, possibly tissue culture effects (Fig. S3).

**Figure 8:**
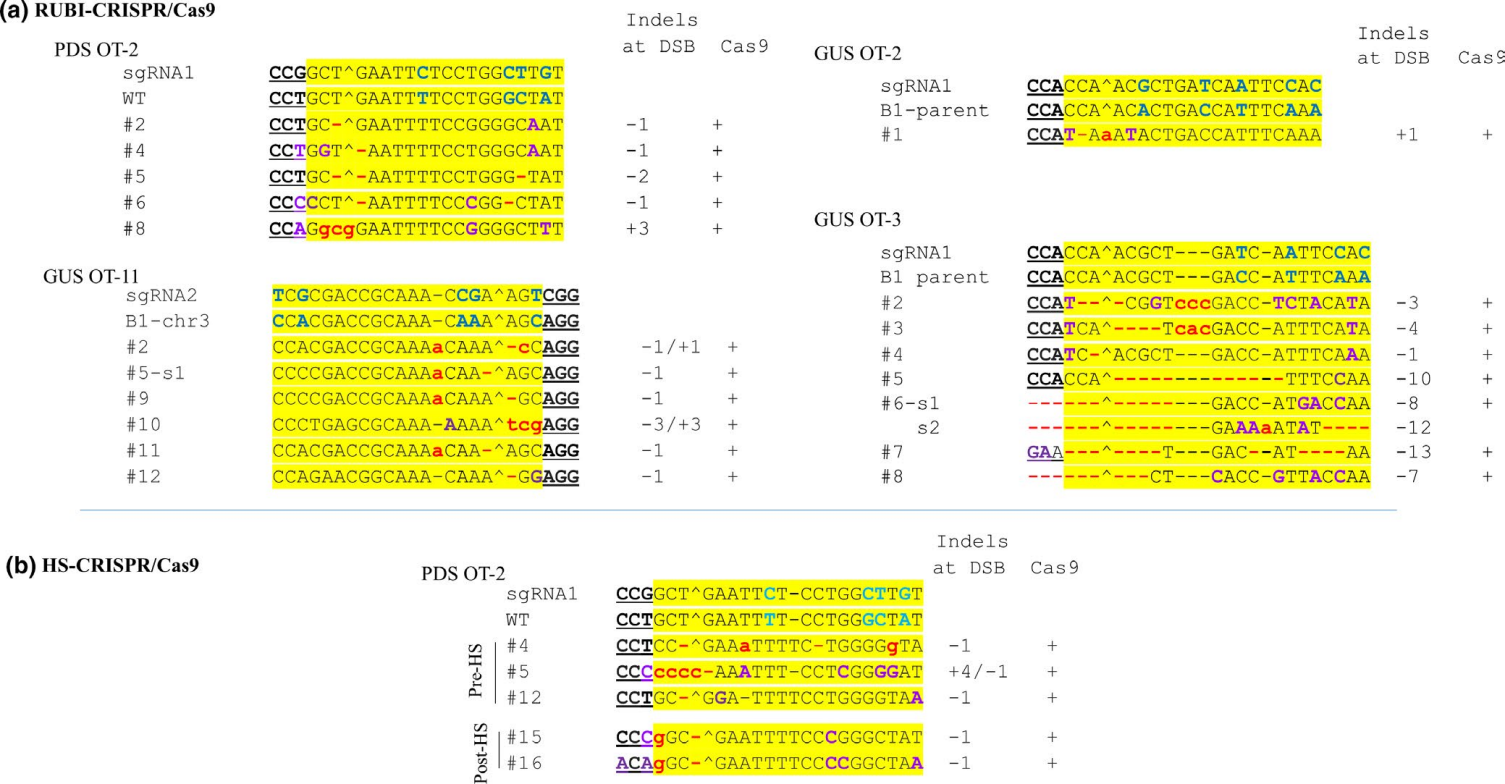
Off‐target site analysis. Sequencing alignments of the predicted *PDS* and *GUS* off‐target (OT) sites in the constitutive (RUBI) and the inducible (HS) CRISPR/Cas9 lines. (a) Sequence alignments of the off‐target sites in the reference (WT or B1 parent) and the RUBI‐CRISPR/Cas9 lines indicating insertion‐deletions (indels) at the predicted DSB sites; (b) alignment of PDS OT2 in HS‐CRISPR/Cas9 pre‐HS and post‐HS lines. Predicted DSB site (^) and PAM (underlined) are indicated. Blue fonts indicate mismatches between the reference sequence and the sgRNA, purple fonts indicate single‐nucleotide polymorphisms between mutant reads and the reference sequence, red dashes are deletion, and red small fonts are insertions. Types of mutations in each line and the Cas9 presence are also shown. The line numbers are given in Table S1 (HS‐CRISPR/Cas9) and Table S7 (RUBI‐CRISPR/Cas9)

Of the four OTs that were evidently targeted by Cas9, PDS‐OT2 was targeted in five of eight RUBI‐Cas9 lines (~62%), showing indels at the predicted DSB site. The remaining three, all of which were GUS‐ OTs, were targeted in 1–7 RUBI‐Cas9 lines (~4–30%) (Fig. 8a, Table 4). Off‐targeting in HS‐CRISPR/Cas9 lines was analyzed in 22 PDS (see Table S1) and 27 GUS samples (see Tables 2, S3, S4), representing pre‐HS or post‐HS samples. Only PDS‐OT2 was found to be targeted among HS‐CRISPR/Cas9 lines, whereas no off‐target mutations were found in GUS‐ OT2, 3 or 11 in any of the HS‐CRISPR/Cas9 lines. Three pre‐HS samples and two post‐HS samples showed off‐target mutations in PDS‐OT2 (Fig. 8b). Mutations in the pre‐HS sample could arise from a high background Cas9 activity or a high transient activity in the progenitor cells during the DNA delivery process. These pre‐HS samples did not contain the on‐target mutations (Table S1). Off‐targeting in the clones lacking on‐target mutations has been reported by others (Aryal, Wasylishen, & Lozano, 2018). In summary, RUBI‐Cas9 was found to be much more active in creating insertion‐deletions in four different off‐target sites, while a reduced rate of off‐targeting was observed in the HS‐Cas9 lines tested in this study.

**Table 4:** Comparative analysis of off‐targeting by the inducible (HS) and the constitutive (RUBI) CRISPR/Cas9 systems

	Off‐Targets (OT)^a^	RUBI‐CRISPR/Cas9			HS‐CRISPR/Cas9			
		Total no. of samples	Samples showing off‐target mutation^b^	%Off‐targeting^c^	Total no. of samples	Samples showing off‐target mutations^b^		%Off‐targeting^c^
						Pre‐HS^d^	Post‐HS^e^	
1	PDS‐OT2	8	5	62.5	22	3	2	22.7
2	GUS‐OT2	23	1	4.3	27	0	0	0
3	GUS‐OT3	23	7	30.4	27	0	0	0
4	GUS‐OT11	23	6	26	27	0	0	0

a From Tables S5 to S6.

b Characteristic insertions‐deletions at the predicted DSB site.

c Percent lines showing off‐target mutations regardless of the heat‐shock treatment.

d Indels detected in room‐temperature samples.

e Indels detected in heat‐shocked samples.

## 4 DISCUSSION

The CRISPR/Cas9 system shows high efficiency targeting in plants and animals, and is often described as a precise system that generates limited or undetectable off‐target effects in plants (Feng et al., 2018; Lee et al., 2018; Tang et al., 2018). However, since the mechanism of targeting is based on a short‐stretch of sequence complementarity and presence of a trinucleotide PAM (NGG) (Jinek et al., 2012), and since mismatches are tolerated at the PAM‐distal end, numerous sites in a complex genome could potentially fall within the scope of CRISPR/Cas9 targeting. Further, sequences ending with noncanonical PAMs such as NAG can also be targeted by Cas9 (Zhang et al., 2014c), and while chromatin structure plays a marginal role in targeting, the secondary structures in the target DNA and the sgRNA could allow significant pairing, in spite of the mismatches at the PAM end (Lin et al., 2014). In both mammalian and plant cells, higher concentrations or the constitutive expression of sgRNA:Cas9 reportedly induced a high rate of off‐target mutations (Hsu et al., 2013; Hu et al., 2018; Pattanayak et al., 2013; Svitashev et al., 2015).

In plants, ribonucleoprotein Cas9 (RNP) has been used as an effective transient expression system (Liang et al., 2017; Svitashev et al., 2016). However, the efficiency of the RNP in plant cells is impacted by the difficulty in delivering it into the cell wall‐bounded compartments and isolating the edited lines in the selection‐free transformation system (Yin, Gao, & Qiu, 2017). Inducible expression systems can be argued as more versatile transient expression systems, provided they generate low or undetectable background expression and a high‐induced expression. Heat‐shock promoters meet these criteria as they have been successfully used in applications where their proper regulation was critical, for example*,* controlling the Cre‐*lox* recombination or the nuclease activity for marker excision (Khattri, Nandy, & Srivastava, 2011; Lloyd, Plaisier, Carroll, & Drews, 2005; Nandy & Srivastava, 2012; Nandy, Zhao, Pathak, Manoharan, & Srivastava, 2015; Zhang et al., 2003).

Here, we describe the use of the heat‐shock (HS)‐CRISPR/Cas9 system consisting of the HS‐inducible expression of the Cas9 and the standard *U3* promoter for sgRNA expression. We found that HS‐CRISPR/Cas9 at the room temperature was suppressed in rice tissue culture and the regenerated plants as mutations in the targeted sites occurred at a low rate in this study (16%). However, upon HS treatment, the characteristic CRISPR/Cas9 mutations were found in ≥ 50% of calli at the targeted sites (Table 1). It is well known that targeting efficiency varies between the genomic sites. However, constitutive CRISPR/Cas9 is often reported to generate ≥80% targeting (Ma et al., 2015; Zhou, Liu, Weeks, Spalding, & Yang, 2014). Therefore, the relative targeting efficiency of HS‐Cas9 with one or two rounds of HS treatments appears to be lower than that of the constitutive‐Cas9. Whether this efficiency could be further improved by additional HS treatments is yet to be determined. The two Cas9 expression systems could not be compared in T0 plants, in this study, as HS‐induced mutations in the plants are evident only as rare or chimeric mutations, indicated by the baseline secondary trace in the sequence spectra (Fig. S1). However, in plants, inheritance rate is the most important criteria of the gene editing efficiency. We show that the HS‐induced mutations in T0 plants were transmitted to the progeny at a high rate and segregated independently from *Cas9* (Table 3). Further, our data reflect on the efficiency of HS‐CRISPR/Cas9 is inducing mutations in the meristem, leading to the mutant cell lineage in the somatic tissue and the germline, which explains the high frequency of one type of mutation observed in the progeny, especially, in the T1 progeny of T0#3 parent (Fig. 7a,b).

Drug‐inducible gene editing systems have been described for the human cells (Dow et al., 2015; Nihongaki, Otabe, & Sato, 2018), but heat‐inducible *Cas9* has so far been used only in *Caenorhabditis elegans* (Li, Yi, & Ou, 2015; Liu et al., 2014). In addition to their potential in curbing off‐target effects, inducible expression systems could confer spatio‐temporal control on gene editing, which can simplify editing of essential genes, avoid lethality by activating *Cas9* at specific developmental stage, and improve gene editing efficiency by inducing *Cas9* in the repair‐competent cells. Use of the heat‐inducible expression system could also leverage improved CRISPR/Cas9 activity by heat‐shock, leading to higher rates of mutagenesis (LeBlanc et al., 2018). Additionally, heat‐shock was found to enhance the sgRNA levels (Fig. S4), which could improve gene editing efficiency, if the sgRNA is limiting. Although, the molecular basis of heat‐induction of sgRNAs is not clear, a similar observation was made in *Arabidopsis* by LeBlanc et al. (2018). Finally, HS‐CRISPR/Cas9 was found to be more precise as it generated either undetectable or a lower rate of off‐target activity on the predicted off‐target sites (Table 4). Of 28 OTs screened in this study, four OTs (PDS‐OT2, GUS‐OT2, 3, 11) were found to be targeted in the constitutive (RUBI‐Cas9) CRISPR/Cas9 lines. Irrespective of the OT site, a higher percentage of off‐targeting was observed in the constitutive RUBI‐Cas9 lines. PDS‐OT2 was targeted in ~62% of RUBI‐Cas9 lines, and GUS‐OTs were targeted in 4–30% of the RUBI‐Cas9 lines. HS‐Cas9 lines, on the other hand, did not show off‐targeting at GUS‐OTs and showed a reduced rate (~22%) of off‐targeting at PDS‐OT2 (Table 4). Since the analysis was based on the Sanger sequencing, off‐targeting in every other line cannot be ruled out; however, this study showed a clear difference in the rates of off‐targeting in the inducible and constitutive CRISPR/Cas9 systems. Finally, as all the clones were derived from tissue culture, base substitutions in the target sites were observed in both HS‐ and RUBI‐CRISPR/Cas9 lines.

In summary, we demonstrate HS‐inducible CRISPR/Cas9 system is generally suppressed at the ambient room temperature in rice, and activated by the heat‐shock treatment. The heat‐shock‐induced genome editing is efficient at producing heritable targeted mutations, while curbing the off‐target mutations. Targeting of more loci and a deeper analysis of off‐targeting will be needed to affirm the precision of the HS‐CRISPR/Cas9 system for wider applications in plant biotechnology. However, this pilot study shows that HS‐CRISPR/Cas9 is a promising genome editing tool that can provide temporal control toward improving the precision of the CRISPR/Cas9 activities. This expression platform could also be used for the temporal control of other gene editing tools such as CRISPR/Cas12a.

## CONFLICT OF INTEREST

Authors state no conflict of interest.

## AUTHOR CONTRIBUTION

VS conceived the idea. BP, SN, and VS participated in experimental design. SZ did rice transformations. SN and BP performed molecular analysis. SN, BP, and VS analyzed the data and drafted the manuscript. All authors read and approved the manuscript.
