# Supplementary figures and images for: Heat‐shock‐inducible CRISPR/Cas9 system generates heritable mutations in rice
Source: Plant Direct. 2019 May 29;3(5):e00145. doi: 10.1002/pld3.145 (PMC6603394; doi:10.1002/pld3.145)

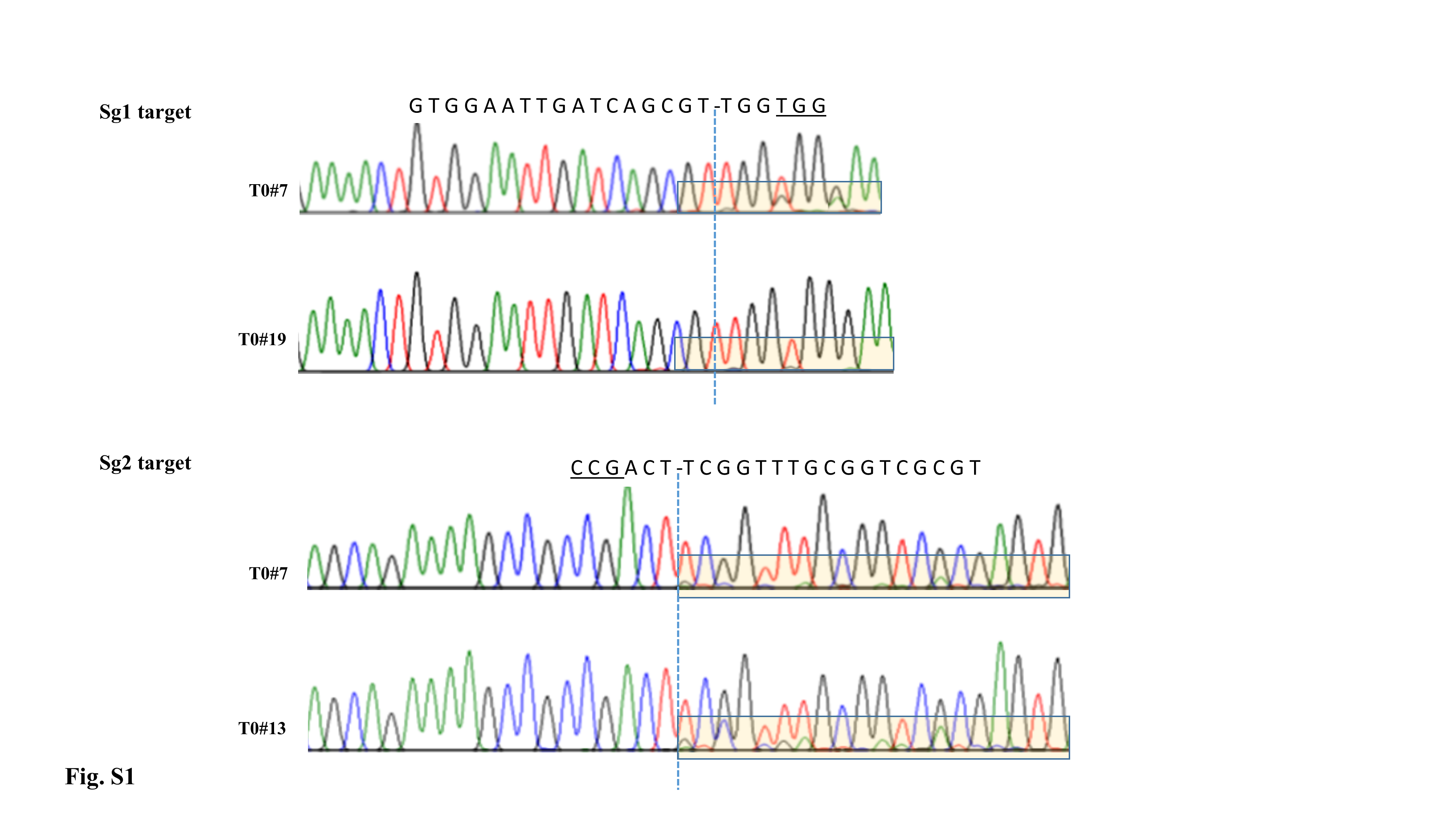

Supplement: Supplementary file 1 [file PLD3-3-e00145-s001.tif]

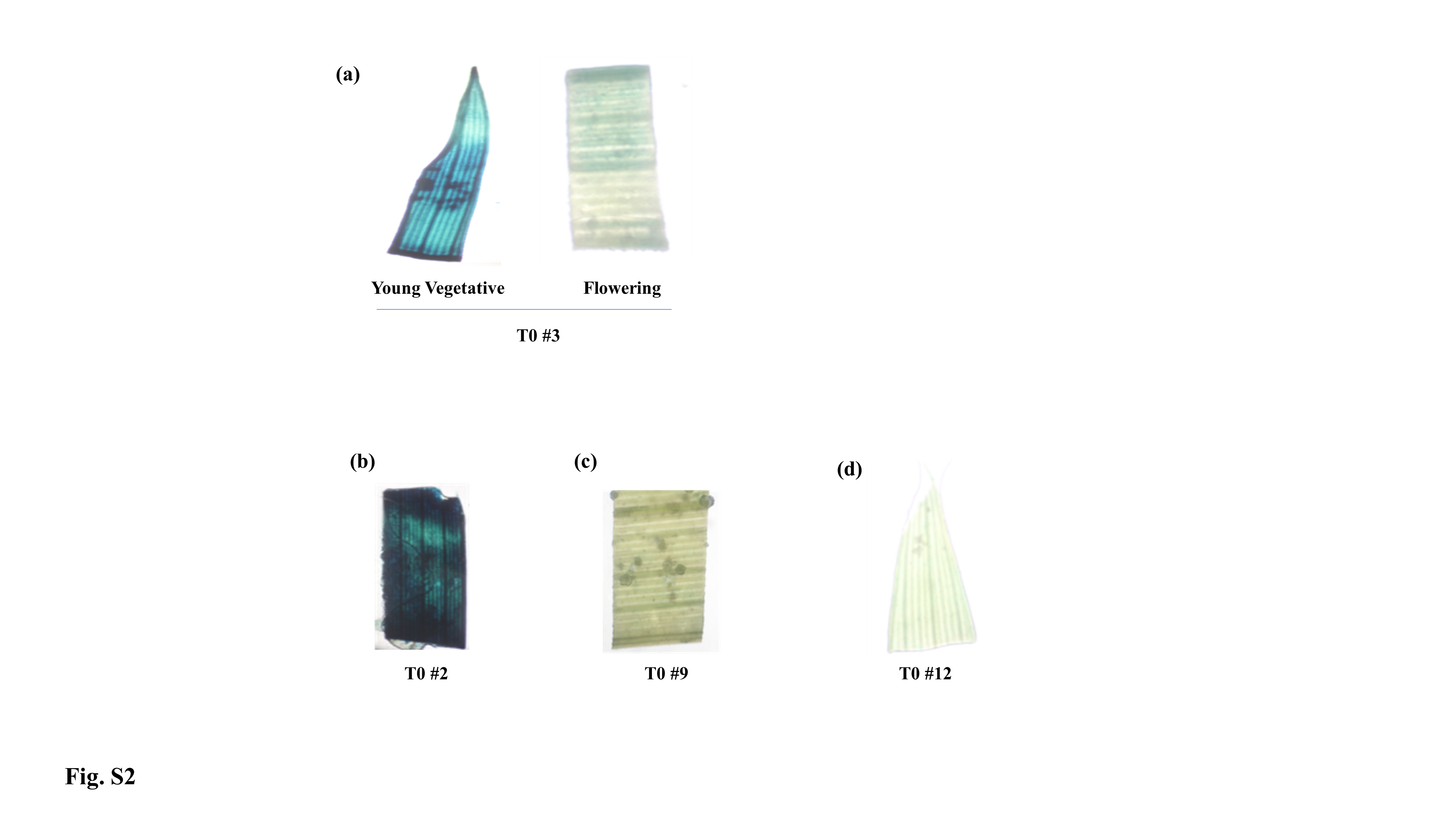

Supplement: Supplementary file 2 [file PLD3-3-e00145-s002.tif]

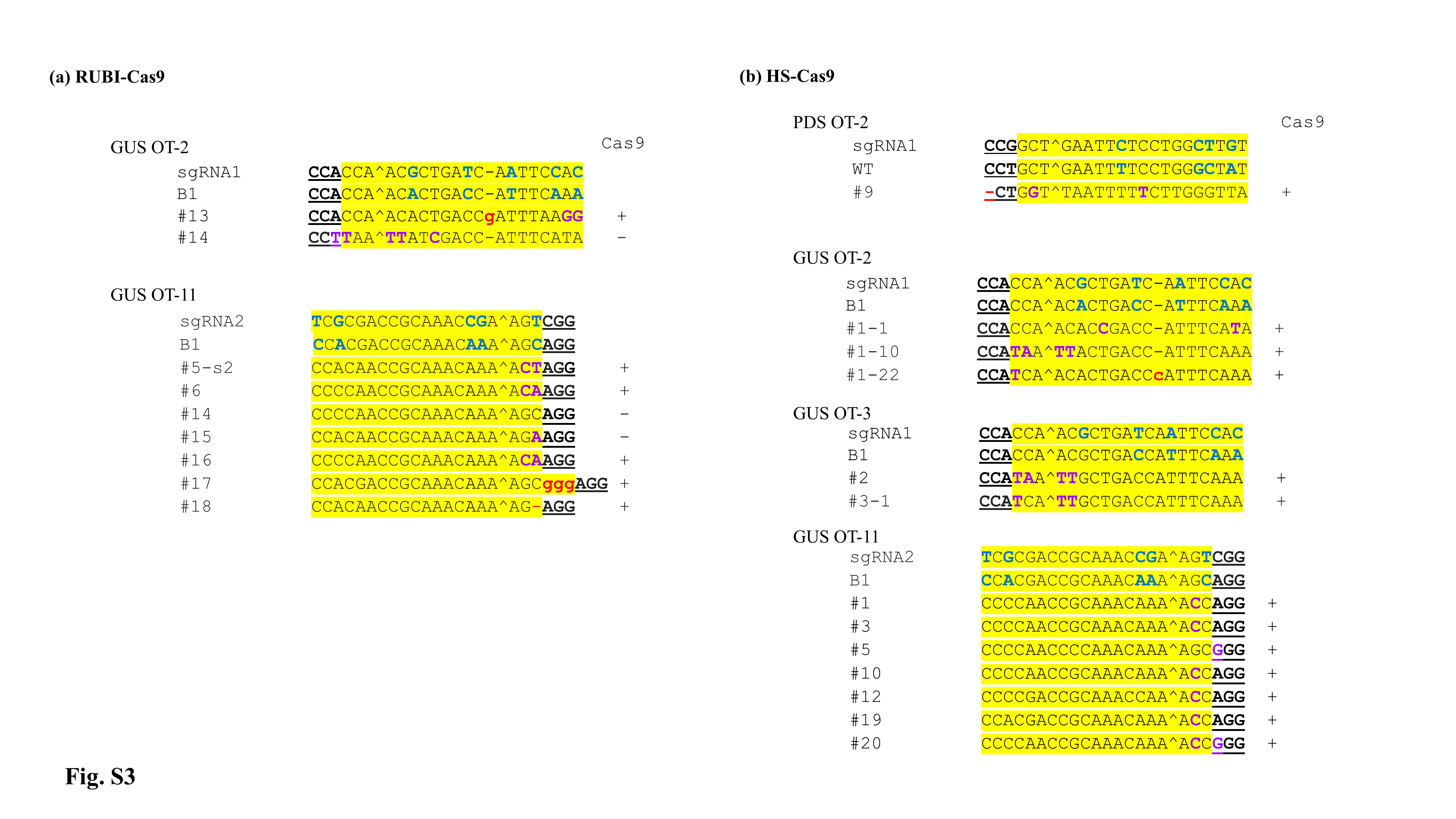

Supplement: Supplementary file 3 [file PLD3-3-e00145-s003.tif]

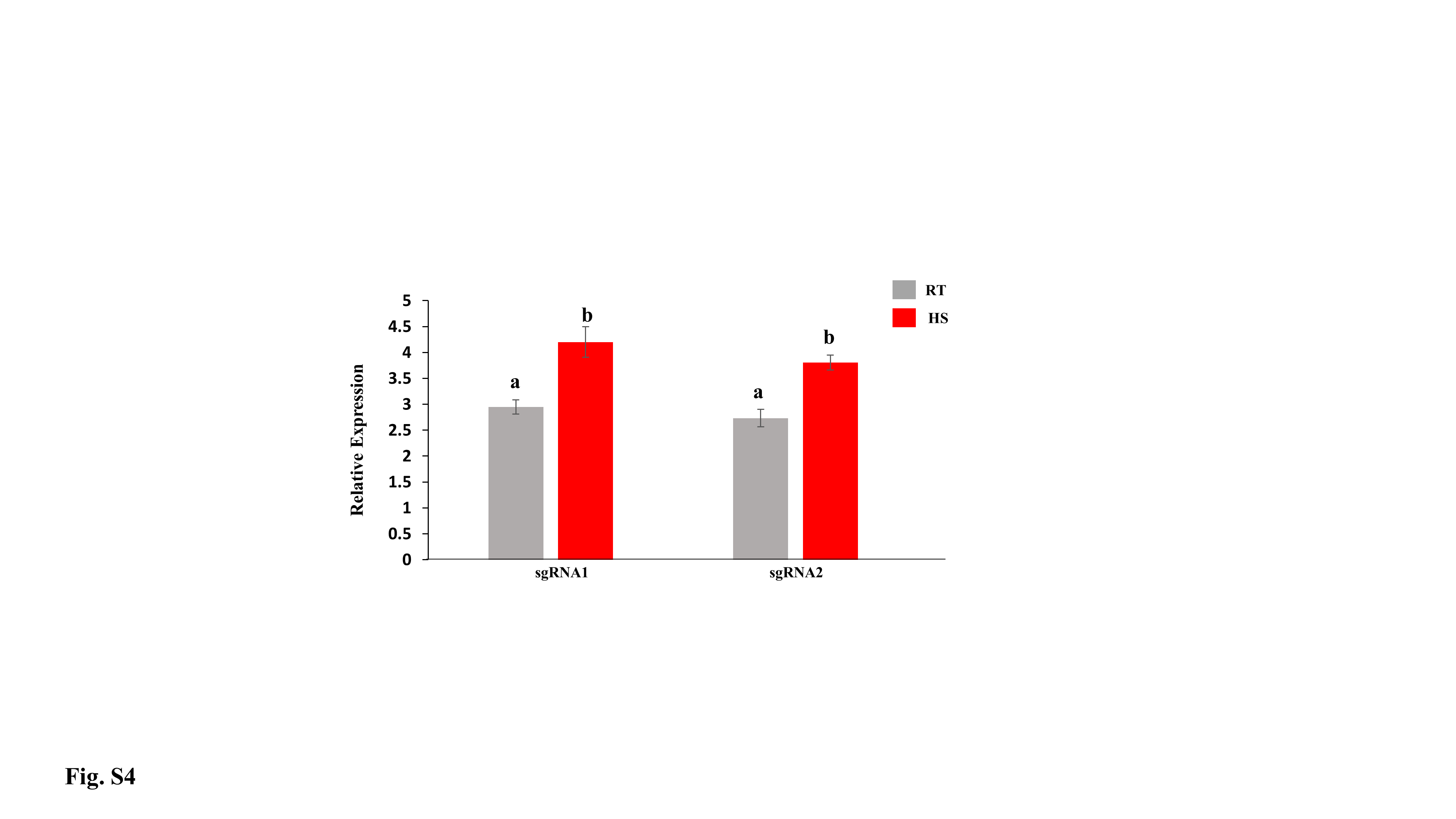

Supplement: Supplementary file 4 [file PLD3-3-e00145-s004.tif]
