# Supplementary material for: Heat‐shock‐inducible CRISPR/Cas9 system generates heritable mutations in rice
Source: Plant Direct. 2019 May 29;3(5):e00145. doi: 10.1002/pld3.145 (PMC6603394; doi:10.1002/pld3.145)
Supplement: Supplementary file 5 [file PLD3-3-e00145-s005.docx]

**Table S1: Heat-shock induced CRISPR/Cas9 targeting of *PDS* gene in rice callus cultures**

|  | **Treatment** | **Sg1** | **Sg2** | **Subject to off-target analysis** | **Off-target mutation** |
| --- | --- | --- | --- | --- | --- |
| 1 | RT | - | WT | Yes | - |
| 2 | RT | WT | WT | Yes | - |
| 3 | RT | Monoallelic^1^ | Monoallelic^1^ | Yes | - |
| 4 | RT | WT | WT | Yes | PDS-OT2 |
| 5 | RT | - | WT | Yes | PDS-OT2 |
| 6 | RT | - | WT | Yes | - |
| 7 | RT | WT | WT | Yes | - |
| 8 | RT | WT | WT | - | - |
| 9 | RT | WT | WT | Yes | - |
| 10 | RT | WT | Biallelic het.^1^ | Yes | - |
| 11 | RT | WT | WT | Yes | - |
| 12 | RT | WT | WT | Yes | PDS-OT2 |
| 13 | HS | - | Mosaic^2^ | Yes | - |
| 14 | HS | Mosaic^2^ | Mosaic^2^ | Yes | - |
| 15 | HS | WT | WT | Yes | PDS-OT2 |
| 16 | HS | WT | WT | Yes | PDS-OT2 |
| 17 | HS | - | Mosaic^2^ | Yes | - |
| 18 | HS | - | Mosaic^2^ | Yes | - |
| 19 | HS | Mosaic^2^ | Mosaic^2^ | Yes | - |
| 20 | HS | WT | Mosaic^2^ | Yes | - |
| 21 | HS | Mosaic^2^ | Mosaic^2^ | Yes | - |
| 22 | HS | WT | - | Yes | - |
| 23 | HS | WT | WT | Yes | - |

^1^Mutations identified by CRISP-ID tool

^2^Multiple overlapping sequencing traces downstream of the predicted DSB sites

**Table S2: Heat-shock induced CRISPR/Cas9**

**targeting of *GUS* gene in rice callus cultures**

| **Samples** | **Treatment** | **Sg1** | **Sg2** |
| --- | --- | --- | --- |
|  |  |  |  |
| 1 | RT | Monoallelic^1^ | WT |
| 2 | RT | WT | WT |
| 3 | RT | WT | WT |
| 4 | RT | WT | WT |
| 5 | RT | WT | WT |
| 6 | RT | WT | WT |
| 7 | HS | WT | WT |
| 8 | HS | WT | Mosaic^2^ |
| 9 | HS | WT | WT |
| 10 | HS | WT | WT |
| 11 | HS | Mosaic^2^ | Mosaic^2^ |
| 12 | HS | WT | Mosaic^2^ |

^1^Mutations identified by CRISP-ID tool

^2^Multiple overlapping sequencing traces downstream of the

predicted DSB sites

| **T1 plant** | **Cas9 PCR** | **GUS staining^1^** | **Sg1 Site** | **Sg2 Site** | **Subject to off-target analysis** |
| --- | --- | --- | --- | --- | --- |
| 1 | + | - | Monoallelic ±1 | Monoallelic +1 | Yes |
| 2 | + | - | WT¶ | WT¶ | - |
| 3 | + | - | WT¶ | WT | - |
| 4 | + | - | WT¶ | WT¶ | - |
| 5 | + | - | WT¶ | Monoallelic +1 | - |
| 6 | - | - | Monoallelic -1 | Monoallelic +1 | Yes |
| 7 | + | - | Monoallelic -2 | WT¶ | - |
| 8 | - | - | Monoallelic -1 | Monoallelic +1 | Yes |
| 9 | + | - | Biallelic (-1/-7) | Monoallelic ±1 | - |
| 10 | - | - | Monoallelic -1 | Monoalellic +1 | Yes |
| 11 | + | - | - | WT | - |
| 12 | + | - | - | Monoalellic +1 | - |
| 13 | + | + | Monoallelic ±1 | - | - |
| 14 | + | - | - | Monoallelic +1 | - |
| 15 | + | - | Monoallelic ±1 | - | Yes |
| 16 | + | - | - | Monoallelic +1 | - |
| 17 | + | - | Monoallelic -1 | - | - |
| 18 | + | + | - | - | - |
| 19 | + | + | - | - | - |
| 20 | - | - | Monoallelic -1 | Biallelic (+1/±3) | - |
| 21 | - | - | - | WT | - |
| 22 | + | + | Monoallelic -1 | - | Yes |
| 23 | - | - | Monoallelic -1 | Monoallelic +1 | - |
| 24 | + | - | - | - | - |

**Table S3: Analysis of T1 progeny of T0#1**

^1^Strong (+) or diminished (-) GUS activity.

^¶^Baseline secondary sequence trace in the sequencing spectra, indicating rare mutations.

| **T1 plant** | **Cas9 PCR** | **GUS staining** | **Sg1 Site** | **Sg2 Site** | **Subject to off-target analysis** |
| --- | --- | --- | --- | --- | --- |
| 1 | + | - | Monoallelic -1 | Monoallelic +1 | Yes |
| 2 | - | - | Monoallelic -1 | Monoallelic +1 | Yes |
| 3 | + | - | Monoallelic -1 | Monoallelic +1 | - |
| 4 | + | - | Monoallelic -1 | Monoallelic +1 | - |
| 5 | + | - | Biallelic homozygous -1 | Monoallelic +1 | Yes |
| 6 | - | - | WT | Monoallelic +1 | - |
| 7 | + | - | Monoallelic -1 | WT | - |
| 8 | + | - | Monoallelic -1 | - | - |
| 9 | + | - | Monoallelic -1 | - | - |
| 10 | - | - | Monoallelic -1 | Monoalellic +1 | - |
| 11 | + | - | Monoallelic -1 | Monoallelic +1 | - |
| 12 | + | - | Monoallelic -1 | Monoalellic +1 | - |
| 13 | - | - | Monoallelic -1 | Monoallelic +1 | - |
| 14 | + | - | WT | Monoallelic +1 | - |
| 15 | + | - | Monoallelic -1 | Monoallelic +1 | - |
| 16 | + | - | Monoallelic -1 | Monoallelic +1 | - |
| 17 | + | - | Monoallelic -1 | Monoallelic +1 | - |
| 18 | - | - | Biallelic homozygous -1 | - | - |
| 19 | + | - | Monoallelic -1 | - | - |
| 20 | + | - | - | Monoallelic +1 | - |
| 21 | + | - | - | Monoallelic +1 | - |
| 22 | + | - | - | - | - |
| 23 | + | - | Monoallelic -1 | Monoallelic +1 | - |
| 24 | + | - | - | Monoallelic +1 | - |
| 25 | + | - | - | Monoallelic +1 | - |
| 26 | + | - | Monoallelic -1 | Monoallelic +1 | - |
| 27 | + | - | Monoallelic -1 | Monoallelic +1 | - |
| 28 | + | - | WT | Monoallelic +1 | - |
| 29 | + | - | WT | Monoallelic +1 | - |
| 30 | + | - | WT | Monoallelic +1 | - |

**Table S4: Analysis of T1 progeny of T0#3**

**Table S5: Potential off-target sites of GUS sgRNAs**

| **Off tar-get site** | **Search criteria** | **Match to** | **Sequence^±^** | **Chromosome: Location** | **Mismatches** | **Off targeting** |
| --- | --- | --- | --- | --- | --- | --- |
| 1 | 20 nt seed | sgRNA1 | CCACCAACGCTGATCtATTaCTa | [1:33226683-33226705](https://plants.ensembl.org/Oryza_sativa/Location/View?r=1:33226681-33226706;tl=pi9CV6i7pis18s2J-18578775-474136280) | 3 | None |
| 2 | 20 nt seed | sgRNA1 | CCACCAAC**a**CTGAcCAtTTCaAa | 7:17045436-17045457 | 5 | Yes^$^ |
| 3 | 20 nt seed | sgRNA1 | CCACCAACGCTGAcCAtTTCaAa | [1:18693750-18693769](https://plants.ensembl.org/Oryza_sativa/Location/View?r=1:18693749-18693769;tl=xBw80h9k96TUp3an-18578776-474136283) | 4 | Yes^$^ |
| 4 | 20 nt seed | sgRNA1 | GTGGtAacGATtAGCGTTGGGGG | 5:23611011-23611033 | 4 | NA* |
| 5 | 12 nt PAM-proximal | sgRNA1 | GATCAGCGTTGGaGG | 12:21835725-21835739 | 1 | None |
| 6 | 20 nt seed | sgRNA1 | GTGGcATTGATCAGCGgTtGTGG | 10:1526410-1526432 | 3 | NA* |
| 7 | 20 nt seed | sgRNA1 | GTaGAAagGATCAGaGTTGGAGG | 8: 23532054-23532076 | 4 | None |
| 8 | 20 nt seed | sgRNA1 | GTGGcAATgTGATCgGCGTTGGTGG | 2:15364552-15364576 | 3 | None |
| 9 | 20 nt seed | sgRNA2 | **ga**GCG**g**CgGCAAACCGAAGTGGG | 4:28810154-28810176 | 4 | NA* |
| 10 | 20 nt seed | sgRNA2 | TCaCAAaCGCAAaCCGAAGGGGG | 2:28847219-28847241 | 4 | NA* |
| 11 | 20 nt seed | sgRNA2 | **c**C**a**CGACCGCAAACC**a**AAG**c**AGG | 3:34663865-34663887 | 4 | Yes^$^ |
| 12 | 20 nt seed | sgRNA2 | TCGC**-**ACCGCAAA**t**CG**t**AG**-**CGG | 2:31797105-31797135 | 4 | NA* |
| 13 | 20 nt seed | sgRNA2 | CCG**c**CTTCGG**c**TTGCGG**c**CGC**-**A | 6:2588632-2588653 | 4 | NA* |
| 14 | 12 nt PAM-proximal | sgRNA2 | GCAAACCGAATGG | 7:19473625-19473638 | 1 | None |

**^±^** Small red fonts are mismatches and red (**-**) dashes are gaps (deletions). PAM are underlined.

**^*^**Not analyzed (NA) due to no/non-specific amplification in negative controls.

^$^Shown in Table 4.

**Table S6: Potential off-target sites of PDS sgRNAs**

| **Off target site** | **Search criteria** | **Match to** | **Sequence^±^** | **Chromosome: Location** | **Mismatches** | **Off -targeting** |
| --- | --- | --- | --- | --- | --- | --- |
| 1 | 20 nt seed | sgRNA1 | AgAAGCacGaAGAATTCAGCTGG | 8:15278101-15278123 | 4 | None |
| 2 | 20 nt seed | sgRNA1 | AtAgcCCAGGAaAATTCAGCAGG | 5:27556570-275565792 | 4 | Yes^$^ |
| 3 | 20 nt seed | sgRNA2 | aAaTGCATttATAACTCATCTGG | 6:14957598-14957620 | 4 | None |
| 4 | 20 nt seed | sgRNA1 | gCAAGCtAGGAtAATTaAGCAGG | 3:7857294-7857316 | 4 | None |
| 5 | 20 nt seed | sgRNA1 | ACAtaCgAGGAGAATTCAGtAGG | 4:31131597-31131619 | 4 | None |
| 6 | 20 nt seed | sgRNA1 | CCTGCTG**-**ATTCT**t**CTGGCTT**c**T | 10:3090619-3090640 | 3 | None |
| 7 | 20 nt seed | sgRNA1 | CCGGCT**tt**ATTCTC**t**T**t**GCTTGT | 1:34782800-34782822 | 4 | None |
| 8 | 20 nt seed | sgRNA1 | ACAAGCCA**a**GATA**t**ATTCAGCAGG | 4:29528959-29528979 | 2 | None |
| 9 | 12nt PAM-proximal | sgRNA1 | GGAGAATTCAGCCGG | 3:34889349-34889363 | 0 | None |
| 10 | 20 nt seed | sgRNA2 | CCTGAT**--**GTTA**c**CCAT**t**CAGTG | 2:15451132-15451152 | 3 | None |
| 11 | 20 nt seed | sgRNA2 | CCTG**c**TGA**c**TT**t**TCCAATGCAGTG | 3:18156090-18156113 | 3 | None |
| 12 | 20 nt seed | sgRNA2 | CCCGATGAG**-**T**-**TCCATGC**t**GTG | 4:29456082-29456102 | 3 | None |
| 13 | 20 nt seed | sgRNA2 | CCTGATGA**-**TTATAC**-**TG**-**AGTG | 11:4868927-4868946 | 3 | None |
| 14 | 20 nt seed | sgRNA2 | CCCGATGAGTTA**c**CCA**-**G**t**AGTG | 12:5686362-5686383 | 3 | None |
| 15 | 12nt PAM-proximal | sgRNA2 | CCTGAT**t**AGTTATCC | 1:39212196-39212210 | 1 | None |

**^±^** Small red fonts are mismatches and red (**-**) dashes are deletions. PAM is underlined.

^$^Shown in Table 4.

**Table S7**: **RUBI-CRISPR/Cas9 lines used in off-target analysis**

| **Gene** | **Line #** | **Tissue**  **Type** | **On-Target mutation^1^** | | **Off-target mutation** |
| --- | --- | --- | --- | --- | --- |
|  |  |  | **Sg1** | **Sg2** |  |
| ***GUS*** | 1 | Leaf | No | No | OT2 |
|  | 2 | Leaf | Yes | Yes | OT3 and 11 |
|  | 3 | Leaf | Yes | Yes | OT3 |
|  | 4 | Leaf | Yes | Yes | OT3 |
|  | 5 | Leaf | Yes | Yes | OT3 and 11 |
|  | 6 | Leaf | Yes | Yes | OT3 |
|  | 7 | Leaf | Yes | Yes | OT3 |
|  | 8 | Leaf | No | Yes | OT3 |
|  | 9 | Callus | Yes | Yes | OT11 |
|  | 10 | Leaf | Yes | Yes | OT11 |
|  | 11 | Leaf | Yes | Yes | OT11 |
|  | 12 | Leaf | Yes | Yes | OT11 |
|  | 13 | Callus | ND | ND | none |
|  | 14 | Leaf | No | No | none |
|  | 15 | Leaf | No | No | none |
|  | 16 | Callus | ND | ND | none |
|  | 17 | Leaf | Yes | Yes | none |
|  | 18 | Leaf | No | Yes | none |
|  | 19 | Leaf | No | No | none |
|  | 20 | Leaf | Yes | Yes | none |
|  | 21 | Leaf | Yes | Yes | none |
|  | 22 | Leaf | Yes | Yes | none |
|  | 23 | Leaf | Yes | Yes | none |
| ***OsPDS*** | 1 | Callus | Yes | Yes | none |
|  | 2 | Callus | Yes | ND | OT2 |
|  | 3 | Callus | Yes | Yes | none |
|  | 4 | Callus | Yes | Yes | OT2 |
|  | 5 | Callus | Yes | ND | OT2 |
|  | 6 | Callus | Yes | ND | OT2 |
|  | 7 | Callus | Yes | ND | none |
|  | 8 | Callus | Yes | ND | none |

^1^Detected either by fragment deletion in PCR indicating dual-simultaneous activity of sg1 and sg2 or by

sequencing of individual targets (data not shown).

ND: not determined

**Table S8: Primers used for the vectors construction and on-site targeted mutagenesis**

| **Primer** | **Sequence (5’ – 3’)** | **Application** |
| --- | --- | --- |
| gGus1-F | TAGGTCTCCTGATCAGCGTTGGgttttagagctagaa | Construction of *GUS* sgRNA1: 5’-GTGGAATTGATCAGCGTTGG-3’ |
| gGus1-R | CGGGTCTCAATCAATTCCACtgcaccagccggg |  |
| gGus2-F | TAGGTCTCCCCGCAAACCGAAGTgttttagagctagaa | Construction of *GUS* sgRNA2: 5’-ACGCGACCGCAAACCGAAGT-3’ |
| gGus2-R | CGGGTCTCAGCGGTCGCGTtgcaccagccggg |  |
| gPDS1-F | TAGGTCTCCCAGGAGAATTCAGCgttttagagctagaa | Construction of  *OsPDS* sgRNA1: 5’-ACAAGCCAGGAGAATTCAGC-3’ |
| gPDS1-R | CGGGTCTCACCTGGCTTGTtgcaccagccggg |  |
| gPDS2-F | TAGGTCTCCATGGATAACTCATCgttttagagctagaa | Construction of  *OsPDS* sgRNA2: 5’-CACTGCATGGATAACTCATC-3’ |
| gPDS2-R | CGGGTCTCACCATGCAGTGtgcaccagccggg |  |
| Ubi1812 | TCTAACCTTGAGTACCTATCTATTA | Genotyping B1 (Ubi:GUS) locus |
| NosR2 | GCGGGACTCTAATCATAAAAACCC |  |
| PDS-F | GGTAGAAATGCCATGCGGGA | Genotyping *OsPDS* locus |
| PDS-R | GTGGTGAGGTTCGGCTGAAT |  |
| Cas9F | AAAGACCGAGGTGCAGACAG | Ca9 genotyping & real-time PCR |
| Cas9R | ACCAGCACAGAATAGGCCAC |  |
| BamH1-Cas9F | CGCGGATCCATGGACTATAAGGACCACGACGG | Construction of HS-Cas9 |
| EcoR1-Kpn1-nosR | GGAATTCGGTACCGATCTAGTAACATAGATGACACCGCCCG |  |
| U3-F | CGGGATCCGACCATGATTACGCCAAGCTTAAG | Construction of sgRNA vector |
| Ptg-R | CGGGATCCAAGCTTTCTAGACCGCCTTGACCCGAATTTGTG |  |
| PDS sg1F | GGC ACAAGCCAGGAGAATTCAGC | Real-time quantitative PCR |
| PDS sg2F | GCACACTGCATGGATAACTCATC |  |
| sgRNA-R | CGA CTC GGT GCC ACT TTT TCA AGT TG |  |
| Ubi-F | CGCAAGTACAACCAGGACAA |  |
| Ubi-R | GCTGTGACCACACTTCTTCTT |  |

**Table S9:** **Primers used in the off target analysis**

| **Gene** | **Off target site** | **Primers** | **Sequence (5’-3’)** | | **Application** |
| --- | --- | --- | --- | --- | --- |
| ***PDS*** | 1 | PDSoff1F  PDSoff1R | TGTGGTTTTGGTTGAGGGCA  GCCCTAAAAGAGGCCGTTCA | | Genotyping PDS off target site 1 |
|  | 2 | PDSoff2F  PDSoff2R | CACTCTTAGCAGTAGGCTATGG  GTAGGAGTTGTACTCACGGATG | | Genotyping PDS off target site 2 |
|  | 3 | PDSoff3F  PDSoff3R | GAGAGGGAGAAACCACACAATC  GCCTCCTGAACTTCTGCTATATTC | | Genotyping PDS off target site 3 |
|  | 4 | PDSoff4F  PDSoff4R | TCAGAGCGATCTCCCAGAAT  TCTTCTCGGGCTCGACCATA | | Genotyping PDS off target site 4 |
|  | 5 | PDSoff5F  PDSoff5R | CAGAGAAGACCACTTACAGA  CACTGACTTACTTCCATCAAGG | | Genotyping PDS off target site 5 |
|  | 6 | PDSoff6F  PDSoff6R | ATACACGCGCCACAGACAAT  ATCGCAGGCGATCTCGAA | | Genotyping PDS off target site 6 |
|  | 7 | PDSoff7F  PDSoff7R | CACTCCATCTCTACACAGCT C  TGTACTGTGACACCGGGTAG | | Genotyping PDS off target site 7 |
|  | 8 | PDSoff8F  PDSoff8R | ACATGGCGTGGTGCATAA  GATTCAGGGATCAGGATGACAC | | Genotyping PDS off target site 8 |
|  | 9 | PDSoff9F  PDSoff9R | CGTCCAATGTATCTCCTCTTC  GCTTGTTGTGGGCTTAGTTG | | Genotyping PDS off target site 9 |
|  | 10 | PDSoff10F  PDSoff10R | CAAAGGACTTACAGGACGTG  TATAGAGAGGGAAGGACCCA | | Genotyping PDS off target site 10 |
|  | 11 | PDSoff11F  PDSoff11R | CCAGTCGAACCATTCAGTGAC  TACAGCCAGAGGTGGTATG | | Genotyping PDS off target site 11 |
|  | 12 | PDSoff12F  PDSoff12R | ACTCCCGACCTCTAGTTTC  CTTGTTGTACGCCTGCAT | | Genotyping PDS off target site 12 |
|  | 13 | PDSoff13F  PDSoff13R | CCAAGTATGCCAAAGGTGTG  GTACGGAGCAAAGTGTTTCC | | Genotyping PDS off target site 13 |
|  | 14 | PDSoff14F  PDSoff14R | GTTTCCGTGCAAATCTGATG  TCTTCGAGCATCCTATCCA | | Genotyping PDS off target site 14 |
|  | 15 | PDSoff15F  PDSoff15R | GCT GAC TAG TGT TAC GTG CA  CAGCACTCACAGCAACATAGC | | Genotyping PDS off target site 15 |
| ***GUS*** | 1 | Gusoff1F  Gusoff1R | | ATGCGCTCGCCATAGAATAG  TCAGCGTGGAAGATGAAGTG | Genotyping GUS off target site 1 |
|  | 2 and 3 | Gusoff2F  Gusoff2R | | CGAAGATTCCTCCGCGATTAC  CATGGATGGAACCAACCTAGAC | Genotyping GUS off target site 2 and 3 |
|  | 5 | Gusoff5F  Gusoff5R | | CCGAACCCATCTTGATTCTCTT  AGAAGAAGCTCCCACCATTTAC | Genotyping GUS off target site 4 |
|  | 7 | Gusoff7F  Gusoff7R | | TCGTACCCGTTCAGTATACGG  GATGACATGCGTCCACAAACAC | Genotyping GUS off target site 6 |
|  | 8 | Gusoff8F  Gusoff8R | | CCTTGTCGTCGTTGGTTCTG  CAAGCGGCACGAGATTTG | Genotyping GUS off target site 7 |
|  | 11 | Gusoff10F  Gusoff10R | | TCGCTGCTCCAAGCTCTC  CAA CAG GTT GCT AGA GCG | Genotyping GUS off target site 10 |
|  | 14 | Gusoff14F  Gusoff14R | | CCCTTCAACACCGGATCGAAG  GAAGAGGCCGGACAGGTTCTT | Genotyping GUS off target site 13 |
