# Supplementary material for: Heat‐shock‐inducible CRISPR/Cas9 system generates heritable mutations in rice
Source: Plant Direct. 2019 May 29;3(5):e00145. doi: 10.1002/pld3.145 (PMC6603394; doi:10.1002/pld3.145)
Supplement: Supplementary file 6 [file PLD3-3-e00145-s006.docx]

**Response to the comments**

Dear Editor,

We have addressed each of your comments and revised the manuscript. In the point by point response below, we have indicated all of the changes made in the manuscript. These changes can be seen in the tracked-change document. The clean version of the revised manuscript is also included.

Sincerely,

Vibha Srivastava

(on behalf of all authors)

**______________**

Comment 1: Please address the comment of reviewer 1 from your Plant Journal submission on article uses ("a" and "the") in your text. Wiley also has an English editing service that you could employ (<https://wileyeditingservices.com/en/english-language-editing/>).

Response: We have reviewed the manuscript and added articles (“a” and “the”) through out the text. These changes are highlighted in the the tracked-change document.

Comment 2: On Page 7, it is written that "Similarly, T0#3 progressively lost GUS activity, while T0#2 that lacked detectable mutations continued to show strong GUS staining. As expected, T0 #9 and #12 that harbored biallelic mutations also did not display GUS staining in the leaves derived from the vegetative or flowering stages of the plant." 
No data are found for these statements. Please remove the statements or provide the data.

Response: The histochemical staining data is summarized in Table 2. We have also added images of the stained leaf cuttings of T0 #2, 3, 9, 12 as the supplemetary data in Fig. S2. To support the statements about histochemical staining with the data, we have referred to Table 2 and Fig. S2 on page 7 and 8.

Comment 3: On Page 8, it is written that "In comparison to these RUBI-Cas9 lines, Cas9 expression was three orders of magnitude lower in HS-Cas9 lines, which could be induced ~100-fold by HS (Fig. 6b; Table 2)."
If I understand Figure 6b correctly, the relative expression before HS is 3 and after HS is 103. This is a 34-fold induction by HS, not 100 folds.

Response: We regret this mistake, and agree that fold-induction is 34 and not 100. We have changed it to 34 fold on page 8.
